# Hanging Drop, A Best Three-Dimensional (3D) Culture Method for Primary Buffalo and Sheep Hepatocytes

**DOI:** 10.1038/s41598-017-01355-6

**Published:** 2017-04-26

**Authors:** Meena Shri, Himanshu Agrawal, Payal Rani, Dheer Singh, Suneel Kumar Onteru

**Affiliations:** Molecular Endocrinology, Functional Genomics and Systems Biology Lab, Animal Biochemistry Division, ICAR-National Dairy Research Institute, Karnal, 132001 India

## Abstract

Livestock, having close resemblance to humans, could be a better source of primary hepatocytes than rodents. Herein, we successfully developed three-dimensional (3D) culturing system for primary sheep and buffalo hepatocytes. The 3D-structures of sheep hepatocytes were formed on the fifth-day and maintained until the tenth-day on polyHEMA-coated plates and in hanging drops with William’s E media (HDW). Between the cultured and fresh cells, we observed a similar expression of *GAPDH, HNF4α, ALB, CYP1A1, CK8* and *CK18*. Interestingly, a statistically significant increase was noted in the *TAT, CPS, AFP, AAT, GSP* and *PCNA* expression. In buffalo hepatocytes culture, 3D-like structures were formed on the third-day and maintained until the sixth-day on polyHEMA and HDW. The expression of *HNF4α, GSP, CPS, AFP, AAT, PCNA* and *CK18* was similar between cultured and fresh cells. Further, a statistically significant increase in the *TAT* and *CK8* expression, and a decrease in the *GAPDH, CYP1A1* and *ALB* expression were noted. Among the culture systems, HDW maintained the liver transcript markers more or less similar to the fresh hepatocytes of the sheep and buffalo for ten and six days, respectively. Taken together, hanging drop is an efficient method for 3D culturing of primary sheep and buffalo hepatocytes.

## Introduction

The liver is a dynamic organ that plays an important role in a variety of physiological processes. The complex functions of the liver include metabolism, storage, excretion, the secretion of plasma proteins, such as albumin, and the detoxification of harmful substances by the enzymes of the cytochrome P-450 oxidases (CYP450s). Liver parenchyma consists 60–80% of hepatocytes^1^. Primary cultures of hepatocytes have been extensively used to study the critical functions of the liver, including the effects of potential toxins on enzymes, metabolism and cellular membranes^2, 3^. Most of the primary hepatocytes are traditionally cultured on two-dimensional (2D) culture systems, in which the hepatocytes are reported to be dedifferentiated and loosing their major functions, like detoxification and plasma protein synthesis^4, 5^. Hepatocytes, being polygonal and multi-polarized in nature, need at least two basolateral and two apical surfaces to interact and exhibit their natural functionality. To provide such an environment, several studies use culture plates coated with the extracellular matrix (ECM) proteins, like collagen, soft collagen, Matrigel, biomatrices^6^ and the derivatives of proteoglycans^7^ on culture plates. Although the liver cultures using these coated plates have advanced the understanding of liver biology to a greater extent, these cultures could not replicate the *in vivo* functions of the hepatocytes^8^. Therefore, three-dimensional (3D) culture systems are required to enhance hepatocyte growth, prolong hepatocyte-specific functions^9^, and to enhance the current understanding of the biologic and toxic response of hepatocytes similar to *in vivo* conditions. In addition, 3D cultures of hepatocytes have a variety of applications ranging from the transplantation or implantation *in vivo* ^10^, screening cytotoxic and pharmaceutical compounds *in vitro* ^11^, the production of biologically active molecules in “bioreactors” and the construction of extracorporeal liver assist device^12^.

In general, 3D cultures provide advantages such as 1) it mimics the *in vivo* cellular microenvironment; 2) the 3D culture can act as a model to study different pathophysiological states; and 3) it is more realistic to grow the cells in 3D to study the effect of drug dosages as the layers of cells and the tight junctions between the cells in 3D cell culture instead of the monolayer culture act as natural barriers, which affect the diffusion of drugs^13^. Furthermore, the gene expression in 3D cultures is much closer to *in vivo* than that of 2D culture systems^14^. Additionally, with the provision of certain biophysical and biochemical signals that affect migration, adhesion, proliferation and gene expression, the 3D culture system promotes certain cellular processes for differentiation and morphogenesis compared to 2D systems^15^. Particularly, the third physical dimension in the 3D culture system allows the orientation of the cell surface receptors to interact with the surrounding cells and extracellular matrix, thus influencing the cellular signaling and gene expression similar to *in vivo* conditions^16, 17^.

Currently, 3D-like or 3D cultures of hepatocytes have been tried using several methods, such as extracellular matrix (ECM) sandwiching, ECM hydrogels, alginate sponges, self-assembly peptide fibers, electrospun fibers, gas foaming scaffolds, 3D printed scaffolds and spheroid cultures. Except the 3D spheroid cultures, all other methods cannot be used for routine toxicity testing due to any of the following issues, like limited 3D organization, mass transfer barriers, poor mechanical properties, and being expensive^1^. Contrastingly, hepatocytes in the scaffold free spheroid cultures are self-assembled into spheres; maintain the direct cell-cell interactions in a multi-polarized fashion and show the liver-specific functionality. Thus, the scaffold-free spheroid cultures are relatively easy to create an ideal platform for routine toxicity testing^1^. Nonadhesive cell culture plates (e.g., poly-2-hydroxyethyl methacrylate (polyHEMA)-coated plates)^15^, bioreactors^17^ and simple hanging drops^18^ can be used for spheroid cultures. However, most spheroid culture studies have employed HepG2 cell-line and not primary human hepatocytes.

Although primary human hepatocyte cultures are gold standard for hepatotoxicity studies, their availability is extremely limited with high inter-donor variability. Therefore, human primary hepatocytes cannot be used for routine toxicity studies and alternative sources of primary hepatocytes are required. Livestock, instead of rodents, could be an alternative source of primary cells because of their resemblance to humans in organ size, variability due to outbred species, and pathogenicity of infectious, metabolic, genetic and neoplastic diseases^19^. In addition, livestock tissues can easily be available from slaughterhouses. On the contrary, tissues from inbred small laboratory rats and mice do not reflect the complex heterogeneous human populations. Further, cattle genome sequencing also revealed that human single copy protein orthologs have more similarity towards the cow protein orthologs than that of the rat and mouse^20^. Specifically, the histological structure of the liver among human, cow, sheep and goat was found to be almost similar^21^. Therefore, livestock tissues could be a great resource for primary cells to model human physiology. However, studies on 3D cell culture for hepatocytes from farm animals, especially ruminants, are limited. Development of 3D cell culture for ruminant hepatocytes is useful not only as *in vitro* models for toxicological studies, but also to understand the physiological processes of hepatocytes in farm animal biology. For instance, the liver plays a major role in handling free fatty acids released from adipose tissue for maintaining body energy requirements as well as high milk production during negative energy balance, which is a common physiological state in early lactating ruminants resulting from high milk yield and low feed intake^22, 23^.Therefore, understanding the liver biology and developing hepatocyte culture systems are urgently needed.

We chose sheep and buffalo as model ruminants in this study, as they are common livestock animals with an easy availability of the tissue samples from slaughterhouses particularly in developing countries, like India. Additionally, these are the closest species to cows, whose genome is similar to humans. But cow slaughter is banned in India. Hitherto, no studies have been conducted on primary 3D cell culture system for primary sheep and buffalo hepatocytes. Therefore, the present work attempts to standardize the spheroid 3D culture system for the primary hepatocytes of the sheep and the buffalo. We used a non-adhesive cell culture technology with poly-HEMA coated plates and hanging drop method to promote spheroid formation of cells unattached to plastic surface. We also employed a 3D-like culture system with a collagen sandwich method that has been used since two decades for hepatocyte culture^24^. Additionally, this study also describes the characterization of the 3D cell culture systems using hepatocyte-specific RNA markers for sheep and buffalo hepatocytes.

## Results

### Success rate of 3D culture systems

The success rate especially regarding the cell viability and integrity for the sheep and buffalo hepatocyte 3D cell culture system was 33% and 20%, respectively.

### Morphological characterization of primary hepatocytes in culture systems

#### Morphology of primary hepatocytes on collagen-coated dishes

Primary sheep hepatocytes formed mainly monolayers on collagen-coated plates and 3D-like structures on collagen sandwich cultures (Supplementary Figure S1). Among various cultures of collagen-coated dishes, primary sheep hepatocytes appeared in 3D-like structures in Hepatozyme-SFM media (CH) on the fifth day. However, these 3D-like structures could not be maintained beyond the fifth day. Therefore, only a monolayer of primary sheep hepatocytes could be seen till the tenth and the twelfth days in CH. Similarly, primary sheep hepatocytes cultured in William’s E medium (CW) also formed only a monolayer with a higher confluence on the fifth day in collagen-coated dishes, but the confluence kept reducing until the tenth and the twelfth days. Although primary sheep hepatocytes formed a monolayer on the fifth day in case of sandwich culture with Hepatozyme-SFM media (SH), they formed 3D-like structures as the culture days went on to the tenth and the twelfth days. A similar pattern was also observed in case of sandwich culture in William’s E media (SW), but with low confluence (Supplementary Figure S1).

Primary buffalo hepatocytes formed into monolayers on collagen-coated dishes (Supplementary Figure S2). Among various cultures of collagen-coated dishes for primary buffalo hepatocytes, 3D-like structures appeared to form on collagen coated dish with Hepatozyme-SFM media (CH) on the third day. But such structures could not be maintained beyond the third day and instead formed into monolayers by the sixth day on CH. Similarly, the buffalo hepatocytes grew in monolayers on collagen-coated plates with Hepatozyme-SFM media containing serum for three days and serum-free but collagen in the media for other three days (CHSC), Williams E media (CW) and Williams E media containing serum for three days and serum-free but collagen in the media for other three days (CWSC). We were not successful in culturing buffalo hepatocytes using collagen sandwich method.

#### Morphology of primary hepatocytes on polyHEMA-coated dish

Among primary sheep hepatocyte cultures in polyHEMA coated-dishes, 3D spheroids were formed on the fifth day with Hepatozyme-SFM media (PH), and they were maintained until the tenth day, but started disintegrating on the twelfth day (Supplementary Figure S3). A similar pattern was also observed for the primary sheep hepatocytes cultured with William’s E media (PW) (Supplementary Figure S3). However, the spheroids were losing their integrity on the twelfth day because of the disturbance caused while changing the media. Among various primary buffalo hepatocyte cultures in polyHEMA-coated dish, 3D spheroids were formed on the third day in Hepatozyme-SFM media containing serum for six days (PHF) (Supplementary Figure S4). However, these structures started disintegrating on the sixth day because of the disturbance caused while changing the media.

#### Morphology of primary hepatocytes in hanging drops

**Figure 1:**
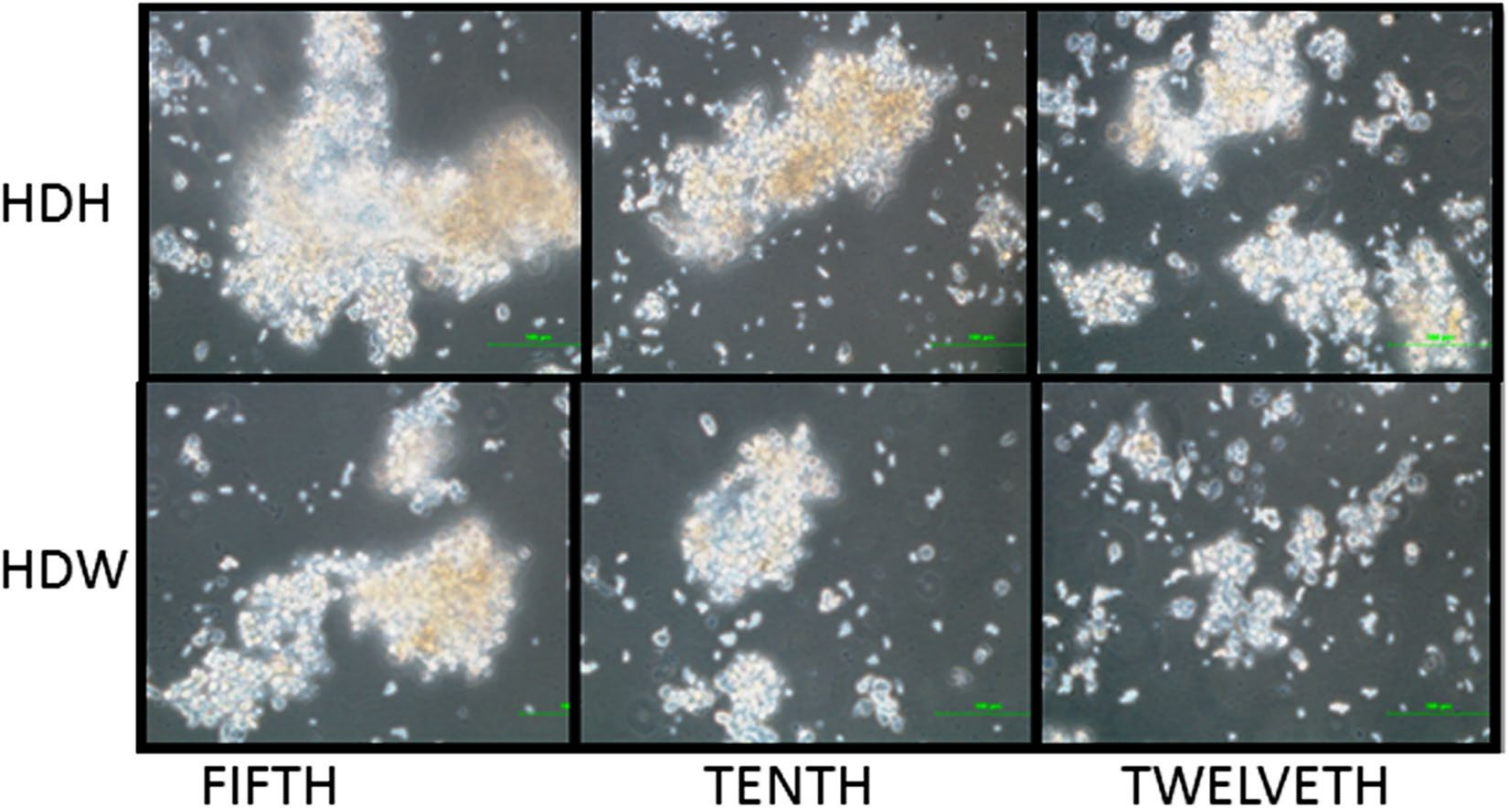
Primary sheep hepatocytes cultured in hanging drops at 200x magnification. Primary sheep hepatocytes formed spheroids in hanging drops with Hepatozyme-SFM (HDH) and William’s E media (HDW) on the fifth day and those spheroids were maintained until the tenth day.

**Figure 2:**
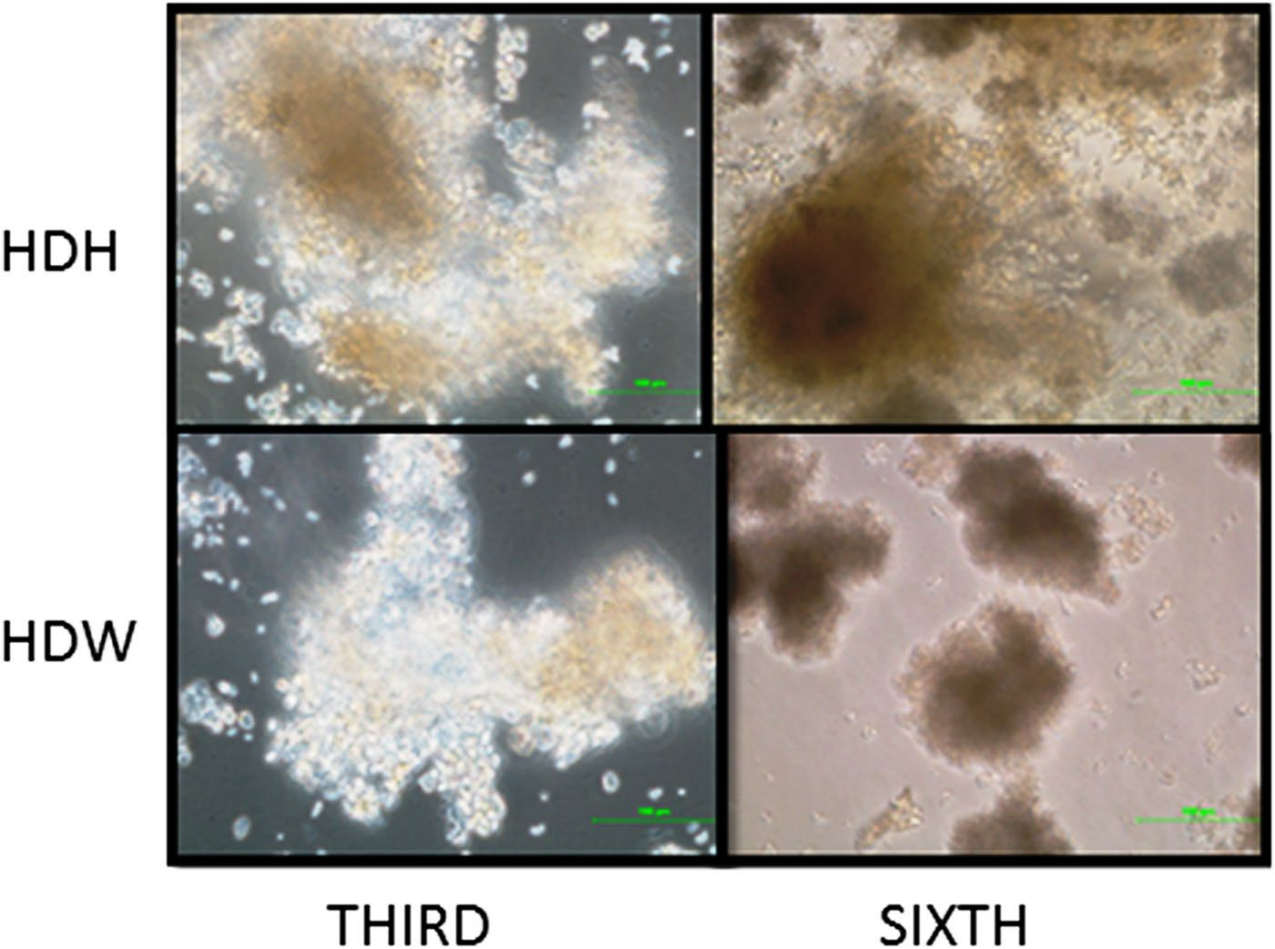
Primary buffalo hepatocytes cultured in hanging drops at 200x magnification. Primary buffalo hepatocytes formed spheroids in hanging drops with Hepatozyme-SFM (HDH) and William’s E media (HDW) on the third day and those were maintained until the sixth day.

The hepatocytes in hanging drops initially spread homogeneously while seeding and no aggregates were formed. Spheroids could be seen in hanging drops with Hepatozyme-SFM media and William’s E media by the fifth day, and these spheroid structures could effectively be maintained till the tenth day in the case of sheep hepatocyte culture system (Fig. 1). For primary buffalo hepatocyte culture, 3D spheroids could be seen in hanging drops with Hepatozyme-SFM and William’s E media on the third day with more confluence in the case of Hepatozyme-SFM media. From third to sixth day the spheroids started disintegrating in both Hepatozyme-SFM and William’s E media (Fig. 2).

### Primary hepatocytes’ viability, integrity and metabolic activity in 3D spheroids

#### Viability

Among primary sheep hepatocyte culture systems, the relative viability by MTT assay was high on the fifth day in case of PH, on the tenth day in case of CW, and on the twelfth day in case of SW and PH. This suggests that SW and PH could be considered as better 3D culture conditions until 12 days for primary sheep hepatocytes (Supplementary Figure S5). For primary buffalo hepatocyte 3D culture, relative viability was higher in the case of CH, CW, and PW (Supplementary Figure S6) than other culture systems except in hanging drops.

#### Integrity

**Figure 3:**
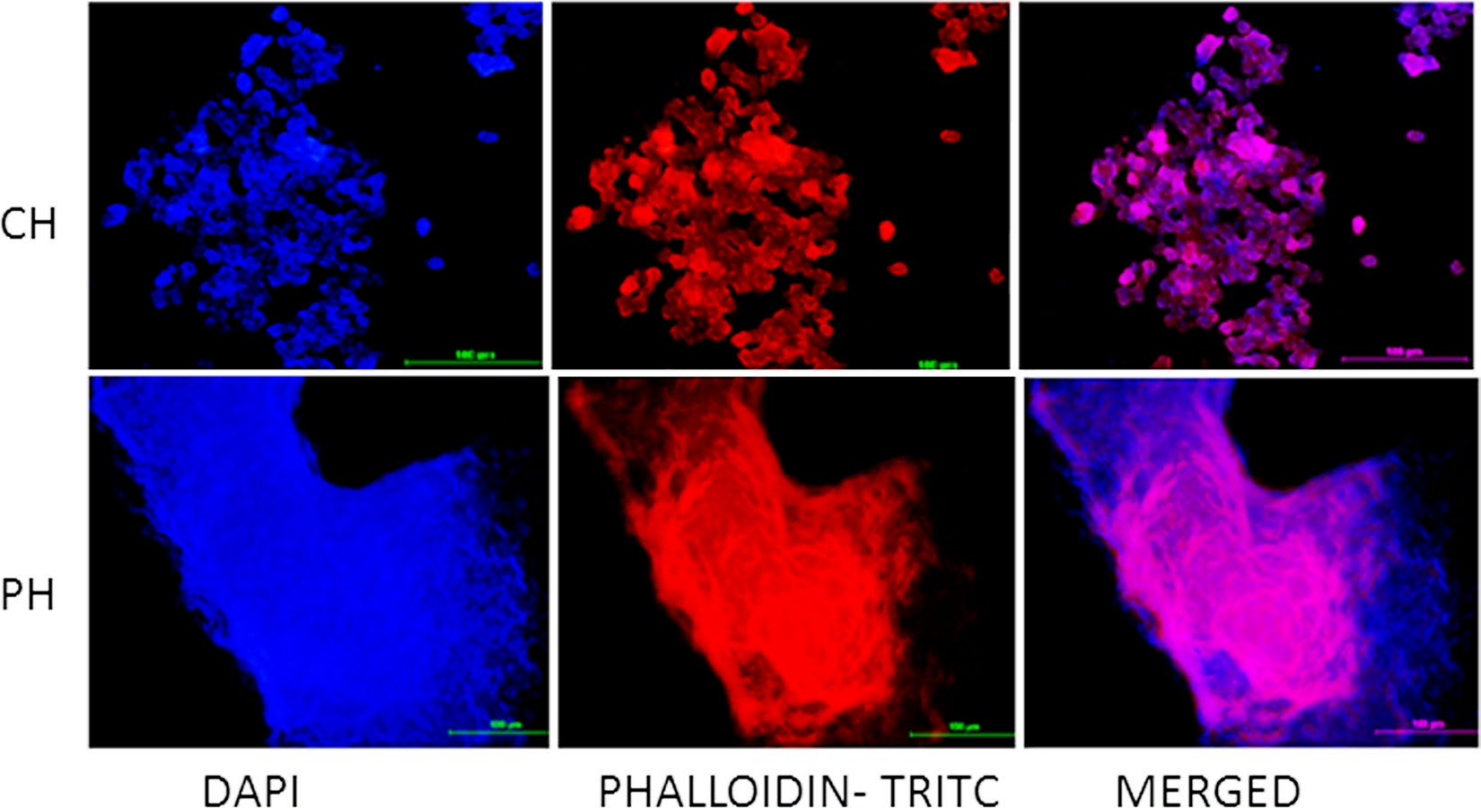
Fluorescent imaging of the primary sheep hepatocytes cultured on collagen-coated and polyHEMA-coated plates on the twelfth day at 200x magnification. The nucleus of the primary sheep hepatocytes were stained with DAPI and the cytoskeletal protein F-actin was stained with Phalloidin-TRITC stain. Hepatocytes formed patches on collagen-coated plates (CH) on the twelfth day of the culture. However, different layers of cells could be observed in the intact spheroids formed on the polyHEMA coated dishes on the twelfth day (PH).

**Figure 4:**
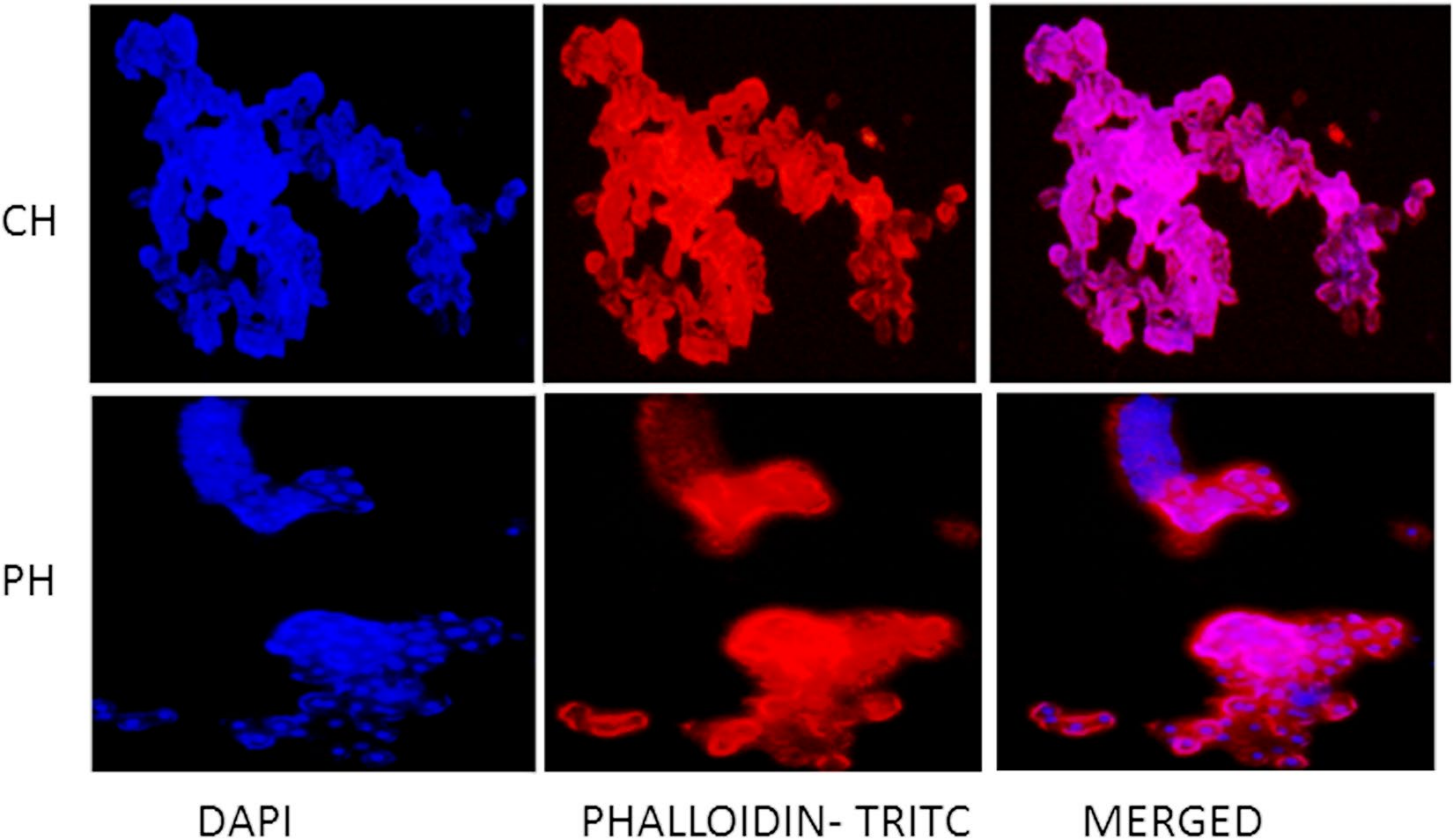
Fluorescent imaging of the primary buffalo hepatocytes cultured on collagen-coated and polyHEMA-coated plates on the sixth day at 200x magnification. The nucleus of the primary buffalo hepatocytes were stained with DAPI and the cytoskeletal protein F-actin was stained with Phalloidin-TRITC stain. Hepatocytes formed patches on collagen-coated plates (CH) on the sixth day of the culture. However, different layers of cells could be observed in the intact spheroids formed on the polyHEMA coated dishes on the sixth day (PH).

Different layers of cells were seen in the 3D structures of the sheep hepatocytes formed in the polyHEMA coated dishes on the twelfth day (Fig. 3) and in 3D structures of the buffalo hepatocytes on the sixth day (Fig. 4). On the contrary, monolayer patches were observed in collagen-coated dishes in the case of sheep hepatocyte culture of the twelfth day (Fig. 3) and buffalo hepatocyte culture on the sixth day (Fig. 4).

#### Metabolic activity

The metabolic activity of the cultured primary hepatocytes was checked by Oilred staining, which stains the neutral triglycerides. Among the sheep hepatocyte culture systems, there was a significant increase (P < 0.05) in adipogenesis in PH and PW on the fifth day, and in SH, PH and PW on the tenth day (Supplementary Figure S5). Among the buffalo hepatocyte culture systems, higher adipogenesis was observed in case of PHF and polyHEMA coated plates with William’s E media containing serum (PWF) on the sixth day (Supplementary Figure S6). Oil red stained images were observed on the twelfth day for the sheep hepatocyte cultures in polyHEMA-coated and collagen-coated dishes (Supplementary Figure S5). Similarly, the Oil red stained images were taken on the sixth day for primary buffalo hepatocyte cultures in polyHEMA-coated and collagen-coated dishes (Supplementary Figure S6).

### Characterization of primary sheep hepatocyte 3D culture systems

**Figure 5:**
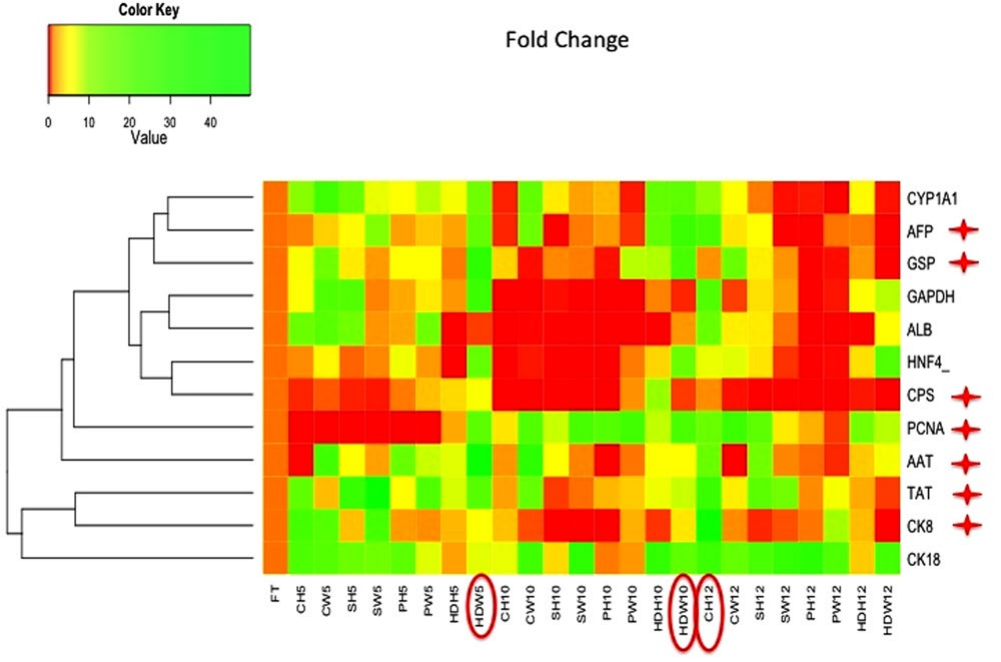
Heat map for the fold change of selected gene markers in different culture systems for primary sheep hepatocyte 3D culture. The expression of the markers, *CYP1A1, GAPDH, ALB, HNF4α*, and *CK18*, was not significantly different between fresh and cultured cells. However, the expression of the markers, *AFP, GSP, CPS, PCNA, AAT, TAT, and CK8*, was significantly (P < 0.05, indicated as ★) different between fresh and cultured cells. The green and red colours indicate higher and lower fold change in the gene expression, respectively, than that of fresh cells. The circled culture systems are considered as best than other culture methods on the fifth, tenth and twelfth days of culture.

A similar expression pattern was observed between fresh and cultured primary sheep hepatocytes for five among eleven typical liver-specific transcript markers (Fig. 5) though there was a high inter-sample variability. Gene expression analysis was performed for fresh primary sheep hepatocytes and those cultured in CH, CW, PH, PW, SH, SW, HDH and HDW on the fifth, tenth and the twelfth day. Specifically, between fresh primary sheep hepatocytes and those cultured in different culture systems, no statistically significant differences were observed in the expression levels of glyceraldehyde 3- phosphate dehydrogenase (*GAPDH*), hepatocyte nuclear factor 4 alpha (*HNF4α*) (Fig. 5 and Supplementary Figure S7), albumin (*ALB)*, cytochrome P450 family 1 subfamily A polypeptide 1 **(** *CYP1A1*) (Fig. 5 and Supplementary Figure S8) and keratin 18 (*CK18*) (Fig. 5 and Supplementary Figure S9). However, there was a statistically significant (P < 0.05) difference in the expression levels of the remaining six selected liver-specific transcripts, such as keratin 8 (*CK8*) (Fig. 5 and Supplementary Figure S9), tyrosine aminotransferase (*TAT*), carbamoyl Phosphate Synthetase 1 **(** *CPS*) (Fig. 5 and Supplementary Figure S10), alpha-fetoprotein (*AFP*), alpha-antitrypsin (*AAT*) (Fig. 5 and Supplementary Figure S11), glucose-6-phosphatase (*GSP*), and a proliferation specific transcript, proliferative cell nuclear antigen (*PCNA*) (Fig. 5 and Supplementary Figure S12). Particularly, there was a statistically significant (P < 0.05) increase in the expression level of CK8 in primary sheep hepatocytes cultured in CH on the twelfth day than fresh hepatocytes. The expression of the *TAT* was significantly increased (P < 0.05) in primary sheep hepatocytes cultured in SH and SW on the fifth day, and CH on the twelfth day than fresh primary sheep hepatocytes. There was a statistically significant (P < 0.05) increase in the expression level of GSP in primary sheep hepatocytes cultured in HDW on the fifth and the tenth day than fresh primary sheep hepatocytes, representing the effective gluconeogenesis process in HDW on the fifth and tenth day of culture system. Similarly, a statistically significant (P < 0.05) increase in the expression level of CPS in primary sheep hepatocytes cultured in HDH on the tenth day than those of fresh cells indicated an increased urea production. AFP is an important marker for undifferentiated cells. A statistically significant (P < 0.05) increased level of this transcript in primary sheep hepatocytes cultured in HDW on the tenth day and CH on the twelfth day than those fresh cells represented the presence of undifferentiated cell population in HDW and CH culture systems. The expression of *AAT* was significantly increased in primary sheep hepatocytes cultured in HDW on the fifth day than fresh cells. As there was a statistically significant increase in the expression level of PCNA, a marker for cell proliferation, in primary sheep hepatocytes cultured in SW, PW and HDW on the tenth day and CW on the twelfth day than those fresh cells. Overall, HDW maintained nine among eleven liver-specific transcripts in cultured primary sheep hepatocytes similar to fresh cells until the tenth day. In addition, this culture system also showed increased levels of the required genes for gluconeogenesis (GSP) and proliferation (AFP and PCNA) of primary sheep hepatocytes. Our data suggest that HDW is a best method for 3D culturing of primary sheep hepatocytes for ten days.

### Characterization of primary buffalo hepatocyte 3D culture systems

**Figure 6:**
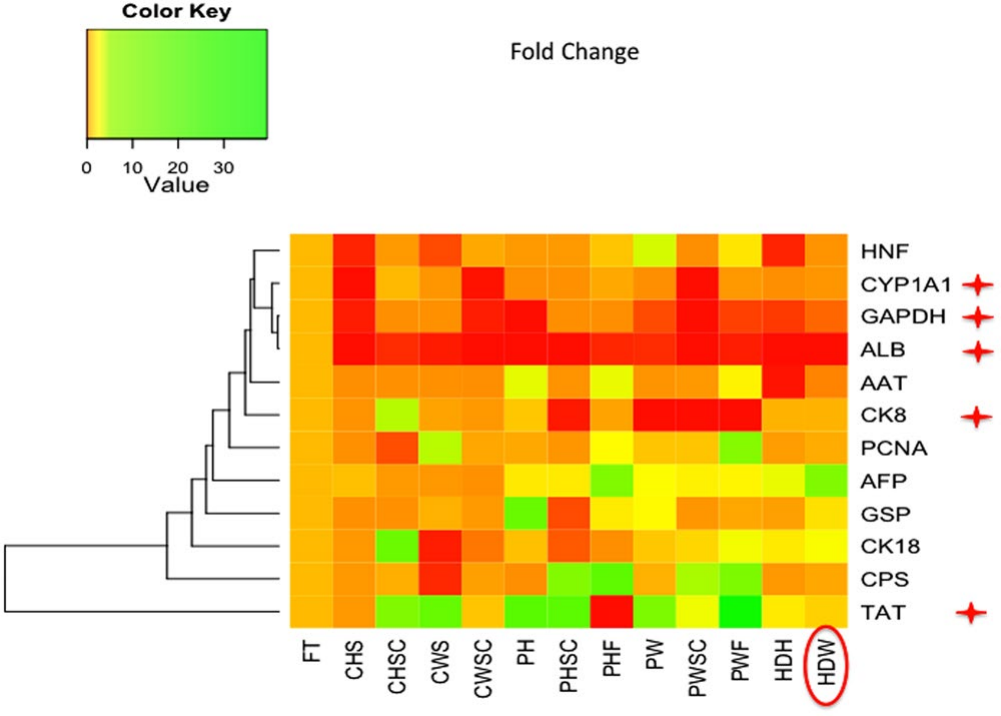
Heat map for the fold change of selected gene markers in different culture systems for primary buffalo hepatocyte 3D culture. The expression of the markers, *HNF4α, AAT, PCNA, AFP, GSP, CK18*, and *CPS,* was not significant between fresh and cultured cells. However, the expression of the markers, *GAPDH, ALB, CK8, CYP1A1*, and *TAT,* was significantly (P < 0.05, indicated as ★) different between fresh and cultured cells. The green and red colours indicate higher and lower fold change in the gene expression, respectively, than that of fresh cells. The circled culture systems are considered as best than other culture methods for culturing buffalo hepatocytes for 6 days in a 3D manner.

Gene expression analysis was performed for eleven typical hepatocyte-specific transcript markers in fresh and cultured primary buffalo hepatocytes in CH, CHSC, CW, CWSC, PH, PHSC, PHF, PW, PWSC, PWF, HDH and HDW culture systems on the sixth day. No significant difference was observed in the expression levels of *HNF4α*, *CPS*, *GSP*, *AFP* (Fig. 6 and Supplementary Figure S13), *AAT*, *PCNA* and *CK18* (Fig. 6 and Supplementary Figure S14) between fresh primary buffalo hepatocytes and those cells cultured in different culture systems. However, there was a statistically significant decrease (P < 0.05) in the expression level of *GAPDH* (Fig. 6 and Supplementary Figure S14) and *ALB* (Fig. 6 and Supplementary Figure S15) in the cells cultured in different culture systems than the fresh cells. Similarly, the expression of the *CK8*, a cytoskeletal protein encoding RNA, was also significantly (P < 0.05) decreased in PWF, PW, PWSC and PHSC than fresh cells (Fig. 6 and Supplementary Figure S15). The expression level of *CYP1A1* in CH, CWSC, PWSC, PH and PW was significantly lower (P < 0.05) than in fresh cells. However, its expression in the CHSC, CW, PHSC, PHF, PWF and HDW was similar to that in fresh cells (Fig. 6 and Supplementary Figure S15). Contrastingly, there was a significant (P < 0.05) increase in the expression of *TAT*, a gluconeogenesis enzyme that catalyzes the L-Tyrosine to p-hydroxyphenylpyruvate in the liver, in PWF than fresh cells (Fig. 6 and Supplementary Figure S15). Overall, the primary buffalo hepatocytes cultured in HDW system maintained nine among eleven selected liver-specific markers similar to those fresh cells. These results indicate that HDW is a best 3D culture system for primary buffalo hepatocytes upto six days.

## Discussion

In this study, 3D culturing of primary sheep and buffalo hepatocytes was performed in collagen-coated plates, collagen sandwich, polyHEMA-coated plates and hanging drop method with an objective to establish a 3D culture system for primary hepatocytes of livestock which are considered as better alternative animal models than rodents. Although 3D culturing of primary hepatocytes was successful in these methods, the success rate in terms of viability was limited to 33% and 20% for sheep and buffalo primary hepatocytes, respectively. This low success rate could be enhanced by avoiding contamination with bacteria and parasites, the impurities of cells during isolation and inappropriate samples at the slaughterhouses. Particularly, selecting the liver samples from healthy young animals of less than two years old without any pathologies and infections would be beneficial for the success of the 3D cultures. The successful cell cultures in the present study were characterized by morphology and eleven hepatocyte-specific transcripts.

Primary sheep and buffalo hepatocytes grew in a monolayer on collagen-coated plates (Supplementary Figures S1 and S2) similar to the established 2D primary hepatocyte cultures of mouse^5^ and human^25^. Although primary sheep and buffalo hepatocytes formed 3D-like structures in Hepatozyme-SFM media (CH) on the fifth and the third day of culture; respectively, the hepatocytes grew in a monolayer in the later culture period. These 3D-like structures during the initial period of cell culture could be formed due to the cell aggregation and attachment by high collagen concentration (17 µg/cm^2^) in collagen-coated plates. Later, the patches of monolayers were formed on the twelfth and the sixth day of culture for primary sheep and buffalo hepatocytes, respectively. This kind of monolayer formation followed by the patchy appearance could be due to reversible dedifferentiation processes of primary hepatocytes on collagen-coated dishes^5^. During the dedifferentiation process, the hepatocytes may enter into proliferation-primed state by the activation of Akt and ERK pathways causing to loose polarity and thus maintain a monolayer state. However, the cells might have grown in patches due to the redifferentiation process at the end of the culture period. Such a basic property of the primary hepatocytes to grow in the form of patches on collagen monolayer was also observed in an earlier study on buffalo hepatocytes^24^. On the contrary, the hepatocytes in collagen sandwich were found to have polarity and apoptotic sensitivity because of the delayed dedifferentiation^1^. Therefore, sheep hepatocytes formed into 3D-like structures rather than monolayers by the tenth day on collagen sandwich (Supplementary Figure S1).

The primary sheep and the buffalo hepatocytes formed spheroids and aggregates (Supplementary Figures S3 and S4) on polyHEMA-coated plates. These plates have a natural property to prevent the cultured cells from adhering on the surface and thus providing a microenvironment for cell-to-cell interactions and communications. This type of culture systems are classified under froth flotation category^9^. Such kinds of spheroids were also observed for primary human hepatocytes on ultra-low attachment culture plates^26^. These primary human hepatocyte spheroids could be maintained initially under serum culture conditions for five days and in serum-free culture conditions for later 30 days^26^. We could not maintain the spheroids of primary sheep and buffalo hepatocytes for more than twelve and six days; respectively, with initial three days under serum and later in serum-free culture conditions. However, the maintenance of the intact spheroids of primary buffalo hepatocytes for six days under 10% serum culture conditions reinforces the significance of serum^27^ to stabilize the spheroids for more than three days. Additionally, the spheroids integrity might be disturbed while changing the media on non-adherent polyHEMA-coated culture plates. Further studies are required to stabilize the sheep and buffalo primary hepatocyte spheroids on polyHEMA coated culture plates for an extended period.

The present study is the first report on hanging drop use for the formation of 3D spheroids of primary hepatocytes as such. Hanging drop method, a scaffold-free system, enables the micro tissue formation without any synthetic materials and force besides the gravity. Hanging drop method was earlier applied to the formation of organotypic cultures of different cell types and cell lines^1^. The time period for spheroid formation typically varies from one cell type to another^28^. The 3D-structures of primary sheep hepatocytes formed in hanging drops (Fig. 1) in the current study were similar to that of chick embryonic liver^28^. According to our observations, the maintenance of the 3D spheroids in hanging drops is challenging. As the sheep and buffalo hepatocyte spheroids in both the Hepatozyme-SFM and William’s E media started disintegrating by the twelfth day and the sixth day, respectively (Figs 1 and 2), the culture period to these hepatocytes was confined accordingly. One of the reasons for the disintegration of the spheroids could be due to disturbances while adding media. The problem can be overcome by using commercially available hanging drop dishes specific to 3D culture system instead of using simple petri dishes as performed in the present study. Another reason for the disintegration of the spheroids might be due to low amounts of extracellular matrix to hold the cells into spheroids by the twelfth and the sixth day for primary sheep and buffalo hepatocytes, respectively, especially under serum free conditions. In addition, the discrepancies observed between the culture periods of the primary sheep and buffalo hepatocytes could be due to either species difference or the differences in the sample collection timing at the slaughterhouses and their transportation.

In addition to the morphological characteristics of primary sheep and buffalo hepatocytes, the current study also emphasized the relative viability of these hepatocytes in different culture systems. The standard MTT assay is well known to measure the relative viability on the basis of mitochondrial dehydrogenase activities in living cells. The relative viability assessed by standard MTT assay^29^ was statistically similar among different culture systems for sheep and buffalo hepatocytes (Supplementary Figures S4 and S5). This indicates that different culture systems maintained comparable relative viability of primary hepatocytes for a defined culture period. Specifically, the cells in 3D spheroids and collagen sandwich had maintained more than 50% viability of their fresh cells. However, in the present study the MTT assay could not be performed for spheroids in hanging drops cultured in a petridish due to non-feasibility of performing washing steps for a single drop among many drops.

Cell integrity and cell-to-cell communication of spheroids in culture systems could be observed by fluorescent microscopy after staining the cells with DAPI and Phalloidin–Tetramethylrhodamine B isothiocyanate. Phalloidin stains the filamentous actin (F-actin), and DAPI binds strongly AT rich region of DNA and thus stains the nucleus^30^. The primary sheep and buffalo hepatocyte spheroids on polyHEMA-coated plates started disintegrating on the twelfth and the sixth day, respectively. Therefore, these were considered for fluorescent staining not only to represent the cell integrity and cell-to-cell communication in the 3D structure of the spheroids but also for easy handling for fluorescence staining. The layers of the cells without any necrotic foci in the hepatocyte spheroids indicate that these spheroids (Figs 3 and 4) might also structurally be simulating the *in vivo* conditions as observed in human primary hepatocytes spheroids^26^. On the contrary, the fluorescence staining clearly showed the patches of hepatocytes on collagen coated plates for sheep and buffalo hepatocytes (Figs 3 and 4).

In addition to the structural integrity, the functional metabolic activity in terms of adipogenesis was also higher in the spheroids than their counterparts on collagen coated plates (Supplementary Figures S5 and S6). The presence of the neutral lipids in hepatocytes indicates the activation of PPAR-α, a key transcription factor regulating carbohydrate and lipid metabolism as well as the expression of adipocyte differentiation-related protein^31^. PPAR-α is highly expressed in tissues like liver, heart, kidney and skeletal muscle, which have high mitochondrial and peroxisome β-oxidation activities^32, 33^. However, a decrease in adipogenesis in primary sheep hepatocytes on the twelfth day in all the culture systems limited the culture period to twelve days.

A similar expression pattern of five liver-specific RNA markers; GAPDH, *HNF4α*, *ALB*, *CYP1A1* and *CK18;* between the cultured and fresh sheep hepatocytes (Fig. 5) shows that the sheep hepatocytes in the culture system are able to mimic the *in vivo* conditions, in spite of inter-sample or inter-donor variability as in humans^34^. Among these candidate markers analyzed, GAPDH is a ubiquitous enzyme of the glycolytic pathway responsible for the conversion of glyceraldehyde 3-phosphate to D-glycerate 1,3-bisphosphate. HNF4α is required for the development of the liver and controlling the expression of many liver-specific genes and their associated critical metabolic pathways^35^. Albumin, a major serum protein, is synthesized and secreted from liver cells. Its main function is to regulate the colloidal osmotic pressure of blood^36^. CYP1A1 enzyme catalyzes the oxygenation of polycyclic aromatic hydrocarbons (PAHs) and heterocyclic aromatic amines/amides (HAAs), resulting in the formation of chemical carcinogens. This is a major gene involved in detoxification process^37^. KRT18 or CK18 encodes the type I intermediate filament chain keratin 18. Keratin 18 (CK18) together with its filament partner keratin 8 (CK8) is the member of the intermediate filament gene family.

A statistically significant (P < 0.05) increase was observed in the expression levels of the remaining six selected liver-specific transcripts such as *CK8*, *TAT*, *CPS*, *AFP*, *AAT* and *GSP*, and a non-liver specific proliferative marker, *PCNA*. The CK8 typically dimerizes with keratin 18 to form an intermediate filament in simple single-layered epithelial cells. This protein plays a role in maintaining the cellular structural integrity and functions in signal transduction and cellular differentiation. Mutations in this gene have been linked to cryptogenic cirrhosis^38^. TAT is a gluconeogenic enzyme that catalyzes the L-Tyrosine to p-hydroxy phenyl pyruvate in the liver. Increased expression of TAT indicates that the cells are using amino acids as an energy source. The GSP is essential for maintaining glucose homeostasis. CPS-I is a ligase enzyme located in the mitochondria and is involved in the production of urea. AAT is the most abundant circulating serine protease inhibitor (serpin), an acute phase reactant, which protects the tissues from enzymes of inflammatory cells, especially neutrophil elastase^39^. The selected 3D culture systems, especially, for sheep hepatocytes are able to mimic the *in vivo* conditions similar or better than the fresh cells during the culture period by maintaining the cellular structural integrity by higher CK8 expression, gluconeogenesisis by TAT and GSP expression, urea production by CPS expression, cellular protection by AAT and proliferation by PCNA expression (Fig. 5).

Between the fresh and cultured primary buffalo hepatocytes, a similar expression level of *HNF4α*, *CPS*, *GSP*, *AFP*, *AAT*, *PCNA* and *CK18* indicates that the hepatocytes in the selected culture systems were able to mimic *in vivo* conditions similar to fresh cells during the entire culture period, especially regarding these seven RNA markers (Fig. 6). The decrease in GAPDH in the primary buffalo hepatocytes cultured in different systems suggest a lower level of glycolysis, and hence these cultured cells might be utilizing energy from other nutrients or pathways. For instance, an increase in the expression of TAT in primary buffalo hepatocyte spheroids cultured on PWF suggest that they might be using amino acids as an energy source. As a consequence of the utilization of amino acids for energy purpose, some of the synthetic processes might be compromised. Therefore, a lower albumin transcript levels in cultured buffalo hepatocytes was observed (Fig. 6). Such a lower expression of albumin was also observed in the two-dimensional culture of buffalo hepatocytes earlier^27^. The CYP1A1 enzymes catalyze the oxygenation of polycyclic aromatic hydrocarbons (PAHs) and heterocyclic aromatic amines/amides (HAAs) resulting in the formation of carcinogens. Similar or lower expression of *CYP1A1* in the cultured buffalo hepatocytes as compared to fresh cells showed that the buffalo hepatocytes in the culture systems were in a healthy state. The assessment and prediction of CYP450 enzyme induction by xenobiotics is one of the main tasks in early drug development. Therefore, the 3D culture systems used for primary buffalo hepatocytes in this study can be utilized for future xenobiotic studies. Among all the 3D culture systems, HDW appeared to be the best culturing system for buffalo hepatocytes as the spheroids in this system have similar or higher expression of all the selected genes, except albumin (Fig. 6), in comparison to fresh cells. Future studies are needed to promote the albumin synthesis in buffalo hepatocytes in HDW culture system.

Overall, the selection of better 3D culture method for sheep and buffalo primary hepatocytes is based on morphology and the expression of eleven selected liver-specific genes in this study. On the basis of the morphology like the stable maintenance of 3D spheroids for twelve days, similar or an increased trend in the expression of the selected liver-specific transcript markers between fresh and the cultured cells in different selected culture systems, the HDW appears to be the best for five and ten days culture. Similarly, for primary buffalo hepatocytes, a stable maintenance of 3D spheroids for six days of the culture and similar gene expression of all the selected liver-specific transcript markers, except GAPDH and ALB, were achieved in HDW. Taken together, HDW could be the best short-time 3D culture method for primary sheep and buffalo hepatocytes maintaining the spheroids and gene expressions. Additionally, the typical characteristics of the cultured primary sheep and buffalo hepatocytes, such as the patches of monolayer on collagen coated plates, different layers of cells in the spheroid structures, adipogenesity and the sustained expression of liver-specific markers, show their potential to be alternative primary hepatocyte models. Therefore, the 3D culture of primary sheep and buffalo hepatocytes can be used for future routine toxicological screening as the tissues can easily be obtained from local slaughterhouses. Besides, these 3D culture models can also be useful in veterinary medicine for understanding the ruminant liver biology. Although the 3D culture systems developed in the present study are first of their kind in livestock, further improvements are needed to extend the viability and cellular integrity of spheroids for an extended period of culture time.

## Methods

### Sources of Chemicals

Cell culture media and medium supplements were procured from Sigma-Aldrich (USA) or Gibco by Life Technologies (USA). Antibiotics were obtained from Invitrogen by Life Technologies (USA). Ethanol was purchased from Merck Millipore (Germany). All other chemicals were bought from Sigma-Aldrich (USA) unless stated otherwise.

### Preparation of collagen coated cell culture plates

A 258 µl of the collagen-I stock (Sigma collagen Rat tail 1) (3 mg/mL) was diluted in 6 ml of 20 mM glacial acetic acid, which was earlier filtered through 0.22 µm syringe filter, to obtain a concentration of 129.2 µg/ml of collagen-I. 250 µl of this dissolved collagen-1 was pipetted into each well of 24 well cell culture plates. The plates were kept under the Biosafety cabinet-II at room temperature for 1 h. Later, the plates were washed with sterile phosphate buffered saline (PBS) two times and placed under UV light for 30 min for further sterilization. Subsequently, the culture plates were sealed with parafilm either for immediate use or stored at 4 °C for future use.

### Preparation of polyHEMA coated cell culture plates

Poly–HEMA (Poly(2-hydroxyethyl methacrylate)) solution was made at a concentration of 12 mg/ml in 95% ethanol. The solution was kept at 45 °C overnight in a shaking incubator to dissolve the polymer completely. To cover the entire cell culture surface of the culture dish, 0.25 or 0.5 ml of the prepared Poly-HEMA solution was layered in each well of either 24 well culture plates or 6 well culture plates, respectively. Further, the culture plates were kept under Biosafety cabinet-II for drying. After drying, the wells were washed with sterile PBS two times and further sterilized by exposing to UV light for 30 min. Later, the culture plates were either used immediately or stored at 4 °C.

### Collection of liver tissues

The sheep liver tissues were collected from the commercial slaughterhouse, Raj meat shop, Sadar Bajar, Karnal, Haryana, India. The buffalo liver tissues were collected from Meem Agro Food Pvt. Ltd., Shamli, U.P, India. Initial processing of the tissues was done at the location of sample collection. Briefly, the tissues were washed with 70% ethanol for nearly 30 s. Later, the tissues were washed for two times with the normal saline (0.9% NaCl) containing the antibiotics, streptomycin (100 µg/ml), gentamycin (50 µg/ml), penicillin 100 (U/ml) and amphotericin (2.5 µg/ml). After initial washings at the slaughterhouse, the tissues were brought to the laboratory in Lactate Ringer’s Solution (LRS) containing the antibiotics at 37 °C. In the laboratory, the tissues were washed six to seven times with normal saline containing the above antibiotics, and processed for hepatocyte isolation and culture.

### Hepatocyte isolation and counting

The modified Seglen’s method^40^ or a two-step perfusion method was used for hepatocytes isolation. In the first step, the perfusion was done with the perfusion buffer-I (pH = 7.4) at the rate of 20 ml per minute by a sterile syringe. The perfusion buffer-I was a Ca^2+^ and Mg^2+^ free Hanks Balanced Salt Solution (HBSS), containing 33 mM *4-(2-hydroxyethyl)-1-*piperazineethanesulfonic acid (HEPES), 0.5 mM ethylenediaminetetraacetic acid (EDTA), streptomycin (200 µg/ml), gentamycin (100 µg/ml), penicillin (200 U/ml) and amphotericin-B (5 µg/ml). The second perfusion was done with 100 ml pre-warmed perfusion buffer II (pH = 7.4) at a flow rate of 20 ml/min with a sterile syringe. The perfusion buffer-II was a Ca^2+^ and Mg^2+^ positive HBSS with 33 mM HEPES, collagenase Type IV (20 µg /mL), streptomycin (200 µg/mL), gentamycin (100 µg/ml), penicillin (200 U/ml) and amphotericin-B (5 µg/ml). Further, the tissue was incubated in perfusion buffer-II for nearly 30 minutes at 37 °C. Later, the tissue was minced with a sterile surgical blade in a sterile petri dish under a Biosafety cabinet-II. The cells and tissue debris were collected in Ca^2+^ and Mg^2+^ free HBSS buffer (HBSS -ve) containing streptomycin (100 µg/ml), gentamycin (50 µg/ml), penicillin 100 (U/ml) and amphotericin (2.5 µg/ml) in 15 ml centrifuge tubes. The collected cells were washed with HBSS–ve by centrifugation initially at 100 g for 5 min at 4 °C followed by next three washings at 50 g for 5 min each at 4 °C. The supernatant in each washing was discarded and the final cell pellet was suspended in 1 ml of HBSS-ve. A 10 µl of the cell suspension was diluted to 100 ml with HBSS-ve, and 10 µl of the diluted suspension was used for checking the impurity of cells under microscope (Nikon Eclipse TI). If the cells other than hepatocytes were observed, then the cells were washed in percoll gradient (1.06, 1.08, 1.12 g/ml) at 750 g for 20 min at 20 °C to remove all non-liver and non-paranchymal cells. Pure hepatocytes formed a ring at the top of the percoll gradient. Heptocytes were collected from the ring by a pipette into a 15 ml microcentrifuge tubes for further washing with HBSS-ve at 1500 g for 5 min. This final washing step was repeated once again to completely remove the percoll from the cells. The final hepatocyte pellet was suspended in 1–2 ml of the culture medium (William’s E or Hepatozyme-SFM) containing streptomycin (200 µg/ml), gentamycin (100 µg/ml), penicillin (200 U/ml) and amphotericin-B (5 µg/ml). Later, we quantified the viability using the trypan blue exclusion method. The cell viability ranged from 85% to 95% with a recovery of nearly 10^6^–10^7^ hepatocytes/ml. The hepatocytes were seeded at a high density (8 × 10^5^ to 10 × 10^5^ cells/ml) in further culture procedures.

### Three-dimensional culture of hepatocytes

Four types of culture systems; collagen monolayer, collagen sandwich, poly-HEMA and hanging drops; under serum followed by serum free conditions were used for primary sheep hepatocyte culture^41^. For collagen monolayer and polyHEMA, the cells were seeded in precoated wells of the respective culture plates at a density of 8–10 × 10^5^ cells/ml, whereas the seeding density was comparatively high (2–3 × 10^6^ cells/ml) in hanging drop method. High cell density in hanging drop was preferred for the formation of the spheroids and to provide *in vivo* microenvironment to the cells. In hanging drop method, the cells were seeded in the form of 10 µl drops on the inverted lid of the 35 mm petri dish, while the dish contained Ca^+2^ and Mg^+2^ free HBSS buffer supplemented with streptomycin (100 µg/ml), gentamycin (50 µg/ml), penicillin 100 (U/ml) and amphotericin-B (2.5 µg/ml). For collagen sandwich, cell seeding was initially done on a collagen-precoated culture dish and the collagen sandwich was prepared after 24 hours of cell adherence according to the guidelines provided along with Rat Tail collagen I (GibcoA10483–01). Two types of culture media, Hepatozyme-SFM or William’s E, containing 5 mM glutamine, 5 µg/ml insulin, 5 ng/ml EGF, 100 mM dexamethasone, 1 mg/ml BSA, 10 mM sodium pyruvate, streptomycin (200 µg/ml), gentamycin (100 µg/ml), penicillin (200 U/ml) and amphotericin-B (5 µg/ml) were used. Accordingly, the culture types are described in Table 1. The media were changed after 24 h for the first time and every 48 h for the rest of the culture period. The cultures were placed at 37 °C in a humidified incubator under 5% CO_2_ atmosphere throughout the culture period of twelve days.

**Table 1:** Culture systems for sheep hepatocytes.

Culture system	Type of 3D culture	Media	First three days	Next Nine days
Collagen-coated dish with Hepatozyme-SFM media (CH)	17 µg/cm^2^ collagen coated*	Hepatozyme-SFM	Media + 10% FBS	Media
Collagen-coated dish with William’s E media (CW)	17 µg/cm^2^ collagen coated*	William’s E	Media + 10% FBS	Media
polyHEMA-coated plates with Hepatozyme-SFM media (PH)	12 mg/ml polyHEMA coated	Hepatozyme-SFM	Media + 10% FBS	Media
polyHEMA-coated plates with William’s E media (PW)	12 mg/ml polyHEMA coated	William’s E	Media + 10% FBS	Media
Collagen sandwich with Hepatozyme-SFM media (SH)	Collagen sandwich	Hepatozyme-SFM	Media + 10% FBS	Media
Collagen sandwich with William’s E media (SW)	Collagen sandwich	William’s E	Media + 10% FBS	Media
Hanging drops with Hepatozyme-SFM media (HDH)	Hanging drop	Hepatozyme-SFM	Media + 10% FBS	Media
Hanging drops with William’s E media (HDW)	Hanging drop	William’s E	Media + 10% FBS	Media

^*^Indicates that those culture systems are more towards 2D rather 3D.

Buffalo primary hepatocytes were cultured in collagen-coated dish, polyHEMA coated dish and hanging drops. Different types of culture conditions were maintained for each type of culture system and media (Table 2). Media composition and antibiotic concentration were kept same as that of sheep culture.

**Table 2:** Culture systems for buffalo hepatocytes.

Culture system	Type of 3D culture	Media	First three days	Next three days
Collagen coated plates with Hepatozyme-SFM media (CH)	17 µg/cm^2^ collagen coated*	Hepatozyme-SFM	Media + 10% FBS	Media
Collagen coated plates with Heaptozyme-SFM media containing collagen (CHSC)	17 µg/cm^2^ collagen coated*	Hepatozyme-SFM	Media + 10% FBS	Media + 166 µg/ml collagen
Collagen coated plates with William’s E media (CW)	17 µg/cm^2^ collagen coated*	William’s E	Media + 10% FBS	Media
Collagen coated plates with William’s E media containing collagen (CWSC)	17 µg/cm^2^ collagen coated*	William’s E	Media + 10% FBS	Media + 166 µg/ml collagen
polyHEMA coated plates with Hepatozyme-SFM media (PH)	12 mg/ml polyHEMA coated	Hepatozyme-SFM	Media + 10% FBS	Media
polyHEMA coated plates with Hepatozyme-SFM media containing collagen (PHSC)	12 mg/ml polyHEMA coated	Hepatozyme-SFM	Media + 10% FBS	Media + 166 µg/ml collagen
polyHEMA coated plates with Hepatozyme-SFM media containing serum (PHF)	12 mg/ml polyHEMA coated	Hepatozyme-SFM	Media + 10% FBS	Media + 10% FBS
polyHEMA coated plates with William’s E media (PW)	12 mg/ml polyHEMA coated	William’s E	Media + 10% FBS	Media
polyHEMA coated plates with William’s E media containing collagen (PWSC)	12 mg/ml polyHEMA coated	William’s E	Media + 10% FBS	Media + 166 µg/ml collagen
polyHEMA coated plates with William’s E media containing serum (PWF)	12 mg/ml polyHEMA coated	William’s E	Media + 10% FBS	Media + 10% FBS
Hanging drops with Hepatozyme-SFM media (HDH)	Hanging drop	Hepatozyme-SFM	Media + 10% FBS	Media
Hanging drops with William’s E media (HDW)	Hanging drop	William’s E	Media + 10% FBS	Media

^*^Indicates that those culture systems are more towards 2D rather 3D.

### Cell viability by MTT Assay

The viability of fresh and cultured primary sheep and buffalo hepatocytes, except in hanging drops, was checked by MTT assay^29^. MTT (3-(4,5-dimethylthiazol-2-yL)-2,5-diphenyl tetrazolium bromide) solution was prepared at 5 mg/ml in PBS (pH 7.2). The assay was performed on the fifth, tenth and the twelfth day of culture for sheep primary hepatocytes, and on the sixth day of culture for buffalo primary hepatocytes. Fresh cells with equal seeding density were taken as control. After removal of the media from 24 well plates, 275 µl of culture media containing 25 µl of MTT solution was poured in each well for cultured cells and to the fresh cell suspension. The plates were kept in an incubator at 37 °C under 5% CO_2_ and 95% relative humidity for four hours. Later, 150 µl of DMSO was added to dissolve the formazan crystals, and the absorbance was measured by an ELISA plate reader (BioTekEon) at 595 nm.

### Cellular integrity by fluorescent imaging

Cellular integrity and interactions were checked by fluorescent staining of cultured sheep and buffalo primary hepatocytes on the twelfth and the sixth day, respectively, as their viability started decreasing on those days. After removal of the media, the cells were washed with 300 µl of 1x PBS for two times. Cells were fixed with 300 µl of 4% paraformaldehyde for 20 minutes and they were further washed with 300 µl of 1x PBS for two times. Further, the permealization of the cells was done by adding 300 µl of Triton-x-100 for 10 minutes and the cells were washed with 300 µl of 1x PBS for two times. Then, 250 µl of the dye, Phalloidin TRITC (Tetramethylrhodamine B isothiocyanate) solution (10 µl in 1 mL), was added and the cells were incubated at room temperature for 45 minutes. Later, the cells were washed twice with nearly 300 µl of 1x PBS. Subsequently, 250 µl of DAPI (4′,6-diamidino-2-phenylindole) solution (10 µl in 1 mL) was added to each well of 24 well plates. Finally, the cells were washed with 300 µl of 1x PBS for two times and the images were taken under the inverted microscope (Nikon Eclipse TI) by using DAPI (excitation wavelength 340–380 nm) and TRITC (excitation wavelength 540/25 nm) filters.

### Metabolic activity by Oilred staining

Primary sheep and buffalo hepatocytes cultured in different cultured systems, except in hanging drops, were stained with Oil red O stain (Sigma, cat no 00625), which particularly stains the neutral lipids in hepatocytes. The staining was done on fifth; tenth and the twelfth day of culture for primary sheep hepatocytes, and on the sixth day of culture for buffalo hepatocytes. Briefly^27^, the medium was removed from the culture and the cells were treated with 10% formalin for 5 minutes at room temperature. Later, the formalin was replaced with fresh formalin and incubated for an hour. After removal of formalin, the cells were washed with 60% isopropanol for two times and dried under Biosafety cabinet-II. The cells were stained with a working solution of Oil red O stain (1.8 mg/mL) for 10 minutes. The stain was washed with distilled water for three times and 500 µl of distilled water was again added to the wells of culture dishes. Then, the absorbance of the water along with stained cells was measured at 520 nm by an ELISA plate reader (BioTekEon Elisa Reader). Finally, the cells were observed under inverted microscope at 200X (Nikon Eclipse TI).

### RNA isolation and cDNA synthesis

Total RNA was isolated with Trizol (Sigma-Aldrich) method. The RNA was immediately used for reverse transcription (RT)-PCR or stored at −80 **°**C until further use. The RNA was quantified by using the Nano Quant (IMPLEN Photometer). The cDNA synthesis was performed with the First-Strand cDNA synthesis kit (Fermantas, St.Leon-Rot, Germany). The reaction mixture contained 100 ng of total RNA, 1 µl of random hexamer (0.2 µg/µL), 1 µl of oligodT primer and the nuclease free water to the volume of 12 µl. The contents were incubated at 65 **°**C for 10 minutes followed by a two-minute incubation at room temperature. The reagents further added were 4 µl of 5x reaction buffer (250 mMTris-HCl, pH 8.3; 250 mM KCl, 20 mM MgCl_2_, 50 mM DTT), 2 µl of RNase inhibitor (20 IU), 1 µl of dNTPmix (10 mM) and 1 µl of Moloney murine leukemia virus reverse transcriptase (200 IU) to a final volume of 20 µl. The contents were successively incubated at 25 **°**C for 10 minutes, 42 **°**C for 30 minutes and 95 **°**C for 3 minutes. The prepared cDNA was diluted to 10 times and the diluted cDNA was used for real-time PCR.

### Real-time PCR for hepatocyte specific transcripts

Characterization and time-dependent dynamics of the different culture systems were checked by the expression levels of hepatocyte-specific transcripts through RT-PCR. A total of eleven hepatocyte-specific transcripts were selected. Gene-specific primers were used for the amplification of the selected transcripts. With the help of Primer blast software (http://www.ncbi.nlm.nih.gov/tools/primer-blast), the gene-specific primers were designed based on the Bovine mRNA sequences available on the NCBI (National Center for Biotechnology Information) database (Table 3). A volume of 5 µl diluted cDNA was mixed with 5 µl of SsoFast^TM^Evagreen^®^ Super Mix, 1 µl of 5 µM forward primer and 1 µl of 5 µM reverse primer. This resultant 12 µl mixture was incubated in light cycler (BioRad, MJMINI PCR Machine) under the following cycling conditions: 95 °C for 5 minutes, followed by 41 cycles of denaturation at 95 °C for 10 s, annealing at 60 °C for 30 s and extension at 70 °C for 30 s. Further, the contents were finally exposed to 95 °C for 5 s, 65 °C for 1 min, 97 °C for 1 min and final cooling at 40 °C for 30 s to obtain the melt curve peaks for ensuring the amplification and generation of single amplification product. Relative quantification of the liver specific transcript was performed by 2^−ΔΔ*Ct*^ method by considering the ribosomal protein, large PO (*RPLPO*) gene as a house keeping gene and the Ct value of the transcript from the cDNA of the fresh uncultured primary hepatocytes as a control.

**Table 3:** Primers for hepatocyte specific RNA markers.

Accession No.	Gene name	Gene symbol	Primer sequence Forward (5′-3′) Reverse (5′-3′)	Product size in basepairs
XM_006046949.1	Albumin	ALB	AAGGCAACAGAGGAGCAACTG TCAGGGTAGGCTGAGATGCTTG	180
XM_005218656.2	Tyrosine amino transferase	TAT	CGGAAAGGGGAGCAGTCTTT CCATCTGGCCTTCCTGCTTT	150
NM_001076124.2	Glucose-6-phosphatase	GSP	GTCTTGTCAGGCATTGCGGT CTCACACCTTCGCTTGGCTT	186
NM_001015557.1	Hepatocyte nuclear factor-4-alpha	HNF-4α	TTCGCCTCACTTCTTCACCC GAACAGGGATGTGGGACGAG	154
AB060696.1	Cytochrome P-450, family 1, member A1	CYP1A1	CAGGGCGATGATTTCAAGGG GTCTGAGGCAGTGGAGAAACT	156
NM_001034034.2	Glyceraldehyde 3-phosphate dehydrogenase	GAPDH	GTTTGTGATGGGCGTGAACC GGCGTGGACAGTGGTCATAA	151
BC103463.1	Alpha-1 antitrypsin	AAT	GAACTGAAGCCGTTCCTGAAG GCCTCTTTCCCAACTACACT	174
NM_001034262.2	Alpha-fetoprotein	AFP	GGACCTTCCGAGCCATAACTG GACACTCCAGCACGTTTCCT	135
NM_001192258.1	Carbamoyl-phosphate synthase-1	CPS	GGTGGCTTGCTTTGGTGAAG TTCCGTGGCAAAGAGCTTGA	175
NM_001033610.1	Keratin-8	CK8	GCCCGGAGCAACATAGACAA TCTCCATGTCTGTGCGCTTT	178
NM_001192095.1	Keratin-18	CK18	GGATTTCAGTCTTGGCGACG ATTGATCTCCTGCTCCCCAG	170
NM_001034494.1	Proliferating cell nuclear antigen	PCNA	AGGAGGAAGCTGTTGCCATAGAG ACTGTAGGAGACAGTGGAGTGG	103
NM_001012682.1	Ribosomal protein large, PO	RPLPO*	TGGTTACCCAACCGTCGCATCTG CACAAAGGCAGATGGATCAGCCA	142

^*^Endogenous calibrator gene in qPCR.

### Statistical analyses

The gene expression was studied in three biological replicates and each value was an average of a technical duplicate. For buffalo hepatocyte culture, comparison among different culture systems and fresh cells was performed by using one-way analysis of variance (ANOVA) with Tukey’s post hoc test, which compares all pairs of columns under test with a consideration of P value < 0.05 as the significant difference. For sheep hepatocyte culture, comparison among different culture systems and fresh cells was performed by using two-way ANOVA by considering the time and the culture system as two factors, followed by a BONFERRONI post hoc test that compares all pairs of columns under test with a consideration of P value < 0.05 as the significant difference. The statistical analyses were performed using Graph Pad Prism version 5.01 (Graph Pad Software Inc., San Diego, California, USA). In addition, a comprehensive visualization of the mean fold changes of all the selected transcripts for different culture systems was visualized by a heat map, which was prepared by the in-house scripts in the R software.

## Electronic supplementary material

Supplementary information 1
