# Supplementary material for: Hanging Drop, A Best Three-Dimensional (3D) Culture Method for Primary Buffalo and Sheep Hepatocytes
Source: Sci Rep. 2017 Apr 26;7:1203. doi: 10.1038/s41598-017-01355-6 (PMC5430879; doi:10.1038/s41598-017-01355-6)
Supplement: Supplementary file 1 — Supplementary information 1 [file 41598_2017_1355_MOESM1_ESM.pdf]

Supplementary information 1

HANGING DROP, A BEST THREE-DIMENSIONAL (3D) CULTURE METHOD FOR PRIMARY BUFFALO AND SHEEP HEPATOCYTES

Meena Shri, Himanshu Agarwal, Payal Rani, Dheer Singh, Suneel Kumar Onteru*

Molecular Endocrinology, Functional Genomics and Systems Biology Lab

Animal Biochemistry Division

ICAR-National Dairy Research Institute, Karnal, India

***For Correspondence**

Dr. Suneel Kumar Onteru, M.V.Sc., Ph.D.,

Senior Scientist

Molecular Endocrinology, Functional Genomics and Systems Biology Lab

Animal Biochemistry Division

ICAR-National Dairy Research Institute, Karnal, India

Phone: +91-184-2259123

Email: suneelvet@gmail.com

Supplementary information 1

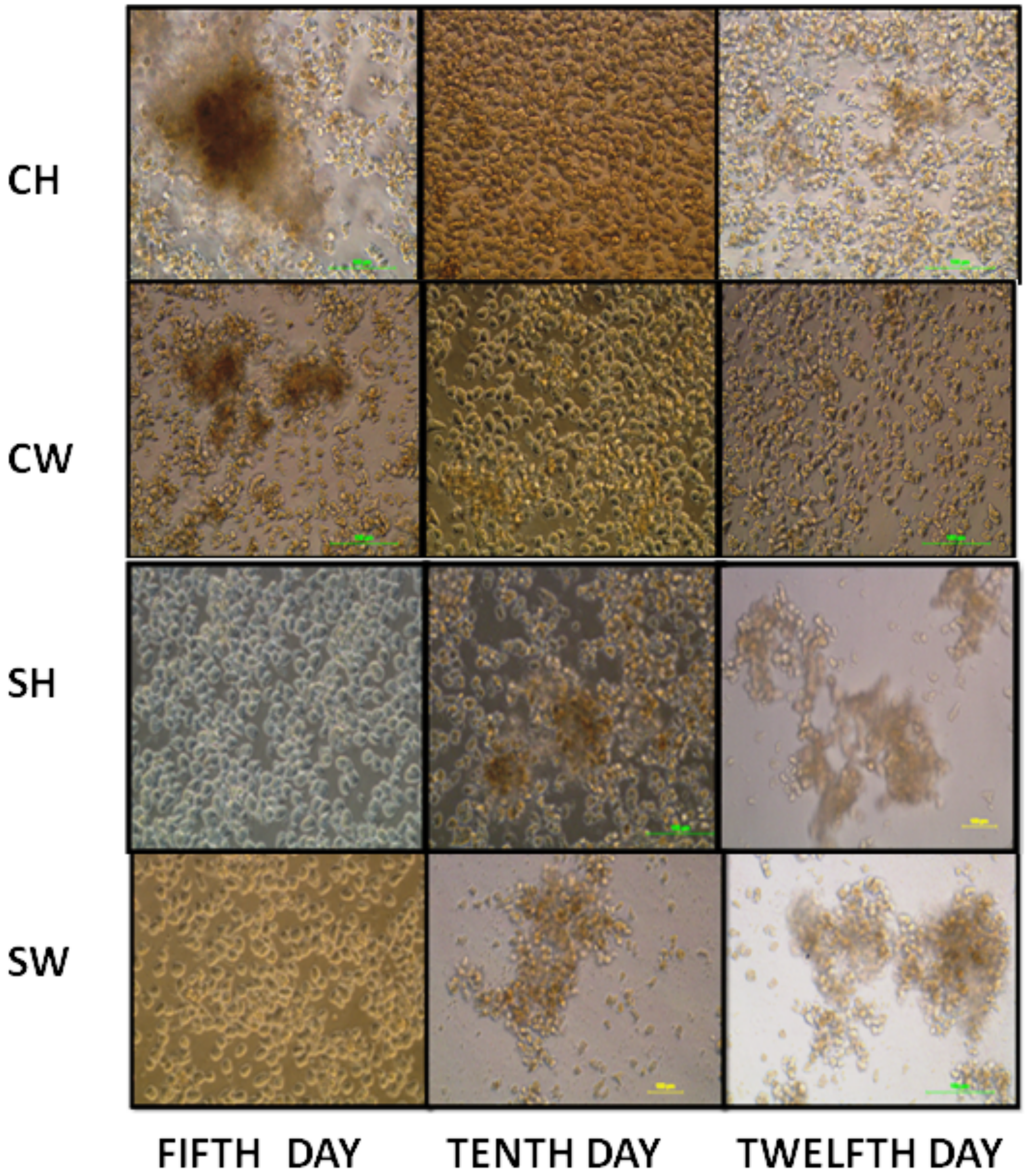

Supplementary Figure S1. Primary sheep hepatocytes cultured on collagen-coated plates and collagen sandwich at 200x magnification. The sheep hepatocytes on collagen coated plates, especially in Hepatozyme-SFM (CH), formed 3D-like structures on the fifth day, a monolayer on the tenth day and a patched monolayer on the twelfth day. A similar pattern can also be observed in William's E media (CW). On the contrary, the hepatocytes cultured in Hepatozyme-SFM (SH) and William's E media (SW) on collagen sandwich showed monolayers on the fifth day and 3D-like structures on tenth and twelfth day.

Supplementary information 1

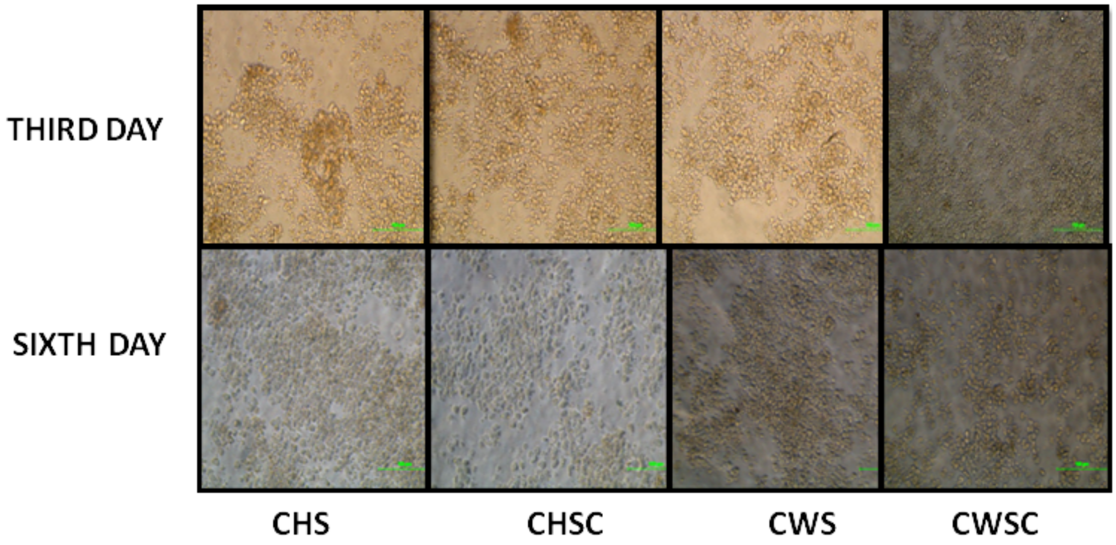

Supplementary Figure S2. Primary buffalo hepatocytes cultured on collagen-coated plates at 200x magnification. Primary buffalo hepatocytes cultured on collagen-coated plates with Hepatozyme-SFM media **(**CHS), Hepatozyme-SFM media containing collagen (CHSC), William's E media (CW) and William's E media containing collagen (CWSC) for six days. The cells in CHS on the third day had higher confluence than other cultures, and this higher confluence was maintained until the sixth day. The cells formed a patched monolayer by the sixth day in these cultures.

Supplementary information 1

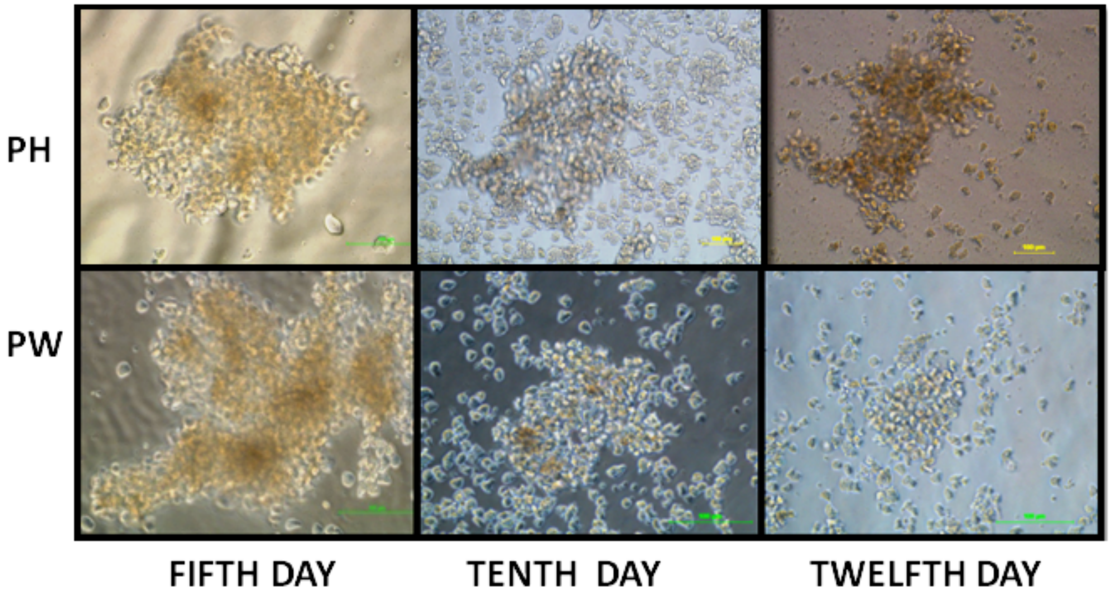

Supplementary Figure S3. Sheep primary hepatocytes cultured on polyHEMA-coated plates at 200x magnification. Primary sheep hepatocytes formed into spheroids on polyHEMA-coated plates in Hepatozyme-SFM (PH) and William's E media (PW) on the fifth day, and those were maintained until the twelfth day.

Supplementary information 1

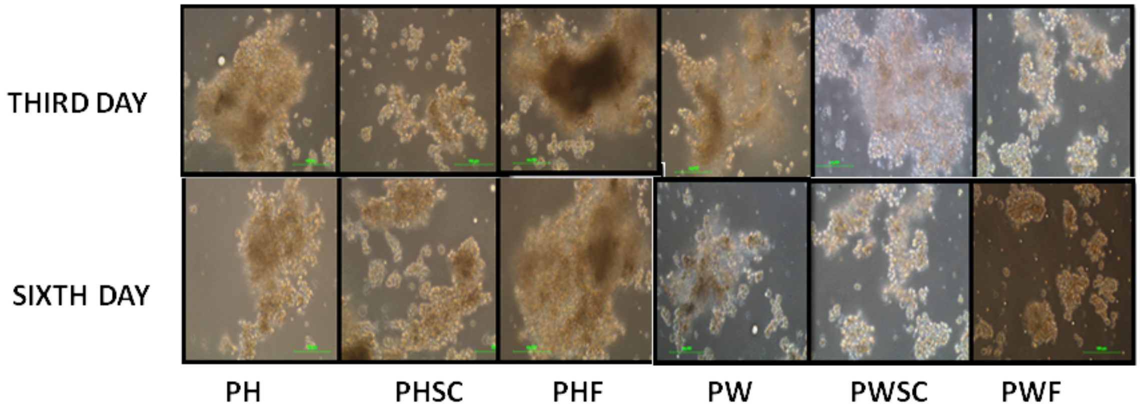

Supplementary Figure S4. Buffalo primary hepatocytes cultured on polyHEMA-coated plates at 200x magnification. Primary buffalo hepatocytes formed spheroids on polyHEMA-coated plates in Hepatozyme-SFM media (PH), Hepatozyme-SFM media along with collagen (PHSC), Hepatozyme-SFM media with 10% FBS (PHF), William's E media (PW), William's E media along with collgen (PWSC) and William's E media with 10% FBS (PWF) on the third day, and those were maintained until the sixth day.

Supplementary information 1

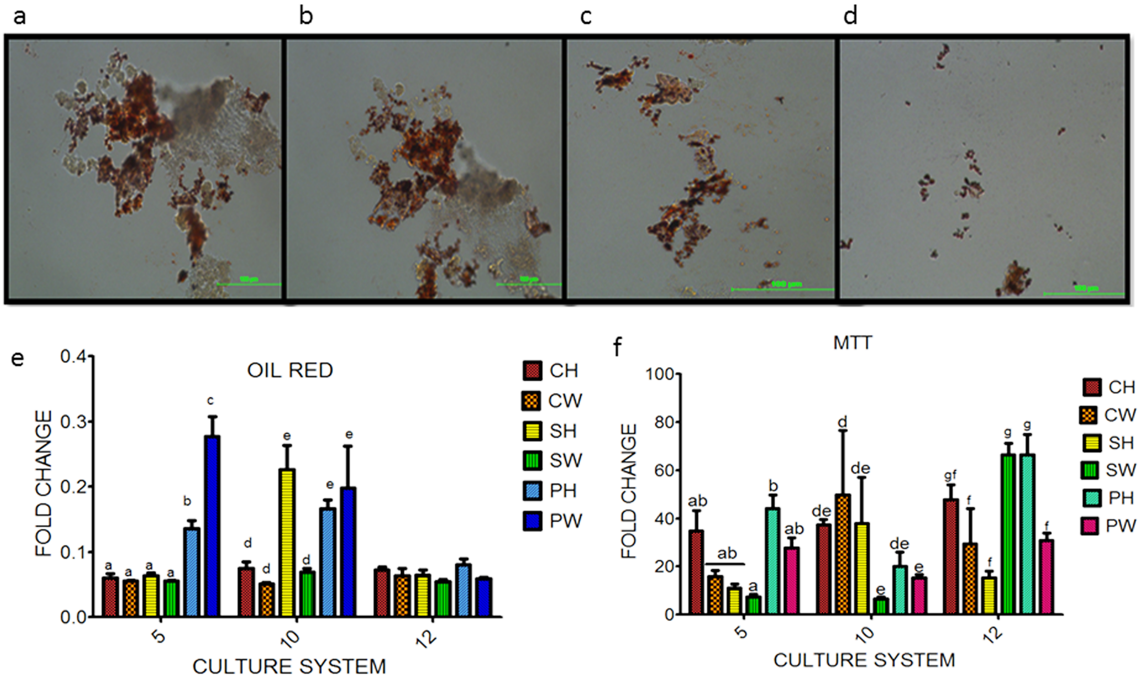

Supplementary Figure S5. Adipogenesis and viability assay for sheep hepatocyte culture. A and B. Oil red stained images taken from two different locations of sheeep hepatocytes in PH at 100X magnification on the twelfth day. **C** and **D.** Oil red stained images taken from two different locations of sheep hepatocyte culture in CH at 100X magnification on the twelfth day. **E.** Oil red staining assay for metabolic activity. The effect of the culture system, time, and their interaction is significant at $P < 0.001$. Each bar represents a mean of three biological replicates and each error bar shows a standard error mean (SEM). The alphabets a,b,c,d and e show significant difference among culture systems with TWO-WAY ANOVA data analysis with BONFERRONI post hoc test at $P<0.05$ on the culture days five, ten and twelve. Adipogenesis is significantly higher in the case of PH and PW on fifth and tenth days. **F.** MTT assay for viability. The effects of the culture system, time, and their interaction are significant at $P < 0.05$, $P < 0.01$ and $P < 0.001$, respectively. The alphbets a, b, c, d, e, f and g show significant difference among culture systems with TWO-WAY ANOVA data analysis with BONFERRONI post hoc test at $P < 0.05$ at the culture days of five, ten and twelve. Viability was significantly higher in case of SW and PH on the twelfth day.

Supplementary information 1

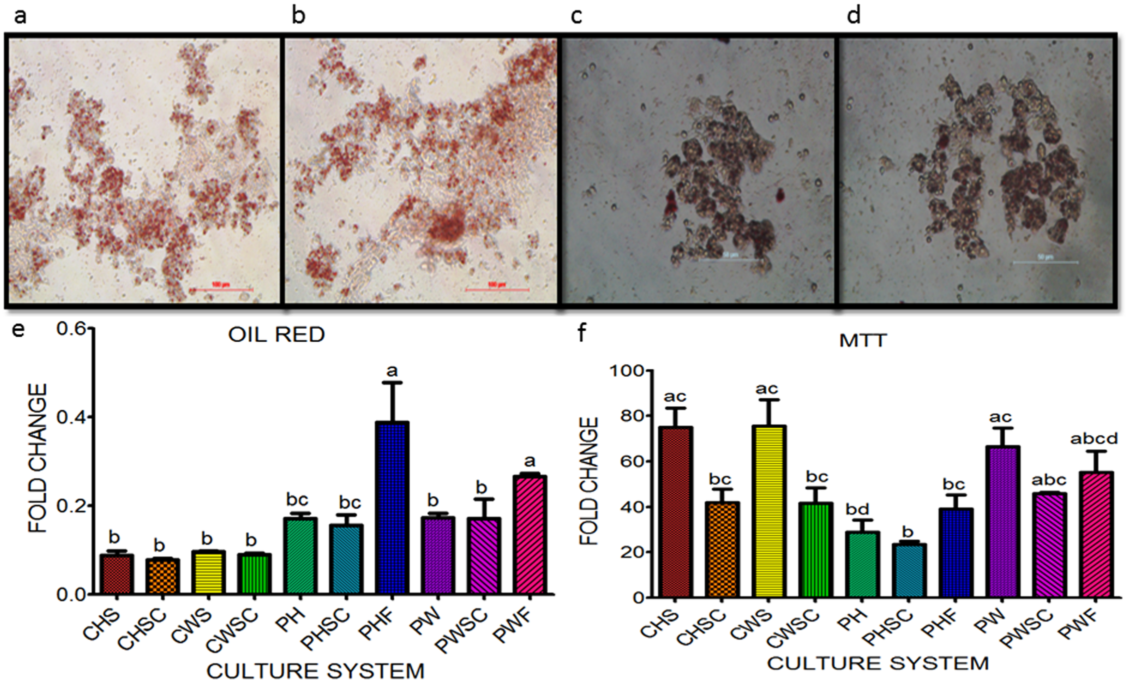

Supplementary Figure S6. Adipogenesis and viability assay for buffalo hepatocyte culture. A and **B.** Oil red stained images were taken from two different locations of buffalo hepatocytes in PH culture at 100X magnification on the sixth day. **C** and **D.** Oil red stained images were taken from two different locations of buffalo hepatocyte culture in CH at 200X magnification on the sixth day. **E.** Oil red staining assay for metabolic activity. Each bar represents a mean of three biological replicates and each error bar shows a standard error mean (SEM). Alphabets a, b, and c show significant difference among culture systems with ONE-WAY ANOVA data analysis with Tukey's post hoc test at $P < 0.05$. Adipogenesis is significantly ($P < 0.05$) higher in the case of PHF and PWF on the sixth day. **F.** MTT assay for viability. Alphabets a, b, c and d show significant difference among culture systems with ONE-WAY ANOVA data analysis for Tukey's post hoc test at $P < 0.05$. Viability was almost equivalent to fresh cells in case of CWS and PW on the sixth day.

Supplementary information 1

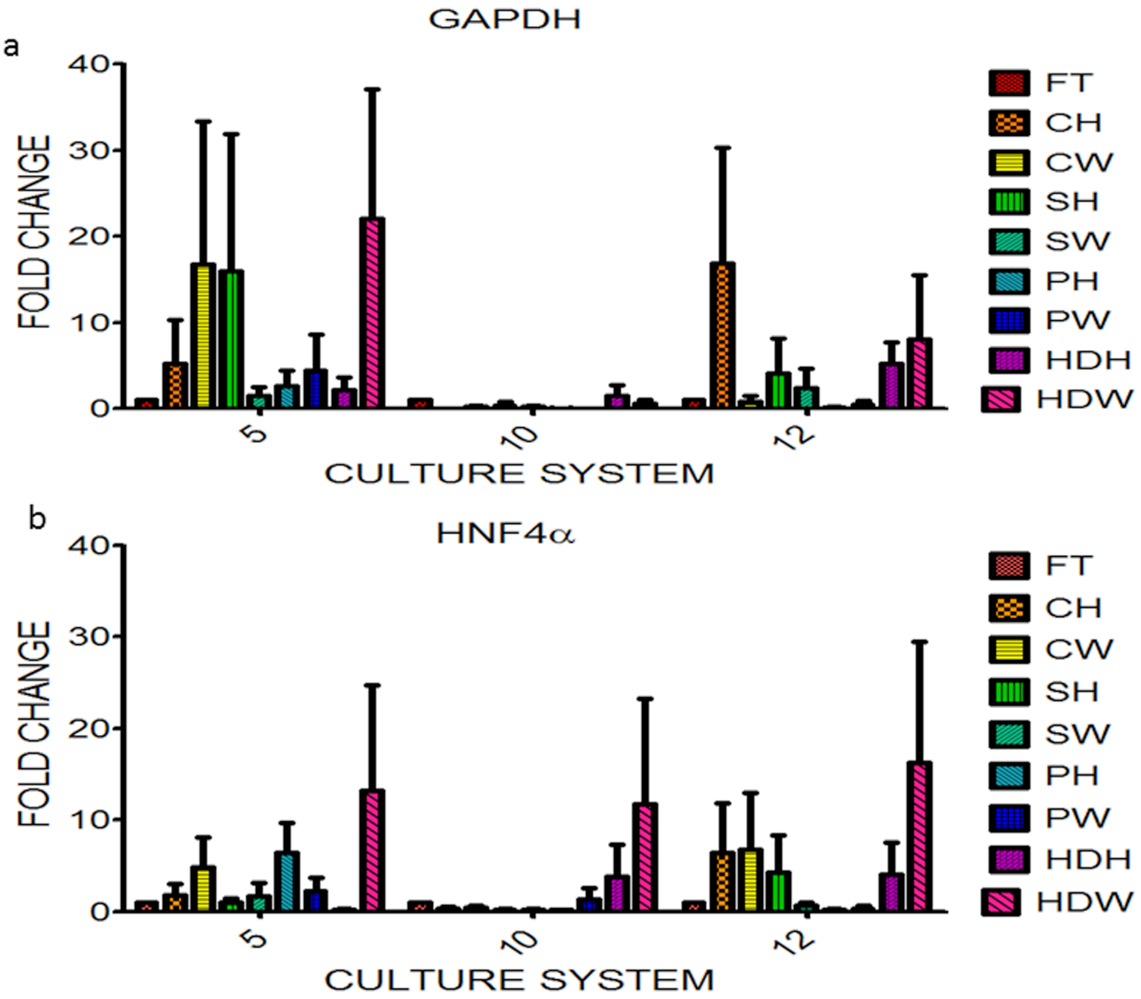

Supplementary Figure S7. Gene expression of the *GAPDH* (Glyceraldehyde 3-phosphate dehydrogenase) and *HNF4α* (Hepatocyte nuclear factor 4 alpha) in cultured primary sheep hepatocytes. There is no significant difference among culture systems by TWO WAY ANOVA data analysis with BONFERRONI post hoc test at fifth, tenth and twelfth day of the culture. **A.** GAPDH. There is a significant ($P < 0.05$) effect of time but not the culture system and the interaction of time and culture. **B.** HNF4α. There is no signficnat effect of culture, time and their interaction. Each bar represents a mean of three biological and two technical replicates. Each error bar shows a standard error mean (SEM).

Supplementary information 1

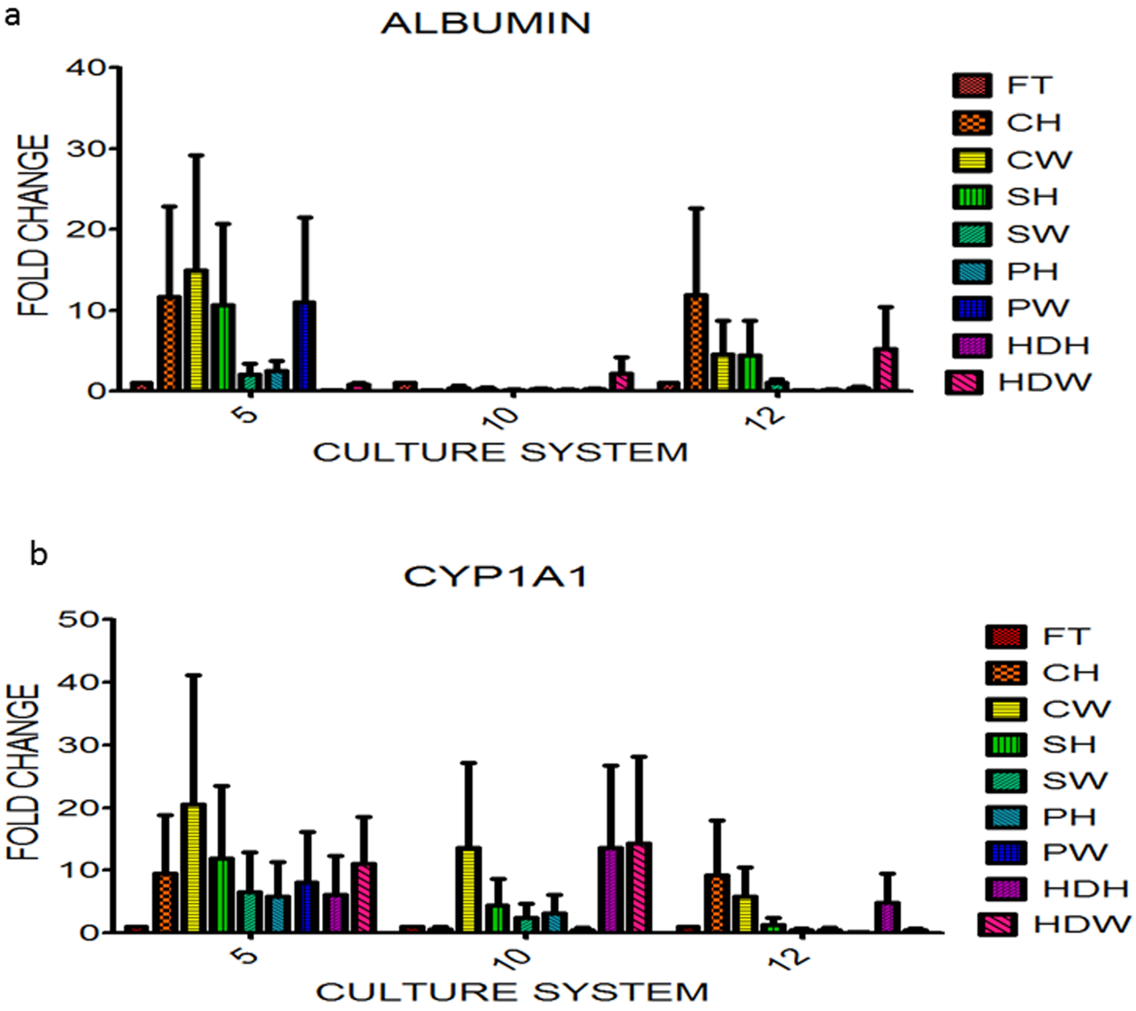

Supplementary Figure S8. Gene expression of the *ALB (Albumin)* and *CYP1A1* (Cytochrome P450, family 1, subfamily A, polypeptide 1) in cultured sheep hepatocytes. There is no significant difference among culture systems by TWO-WAY ANOVA data analysis with BONFERRONI post hoc test at the fifth, tenth and twelfth day of culture. **A.** ALBUMIN, There is a significant ($P < 0.05$) effect of time but not the culture system and the interaction of time and culture. **B.** CYP1A1. There is no signficnat effect of culture, time and their interaction. Each bar represents a mean of three biological and two technical replicates and each error bar shows a standard error mean (SEM).

Supplementary information 1

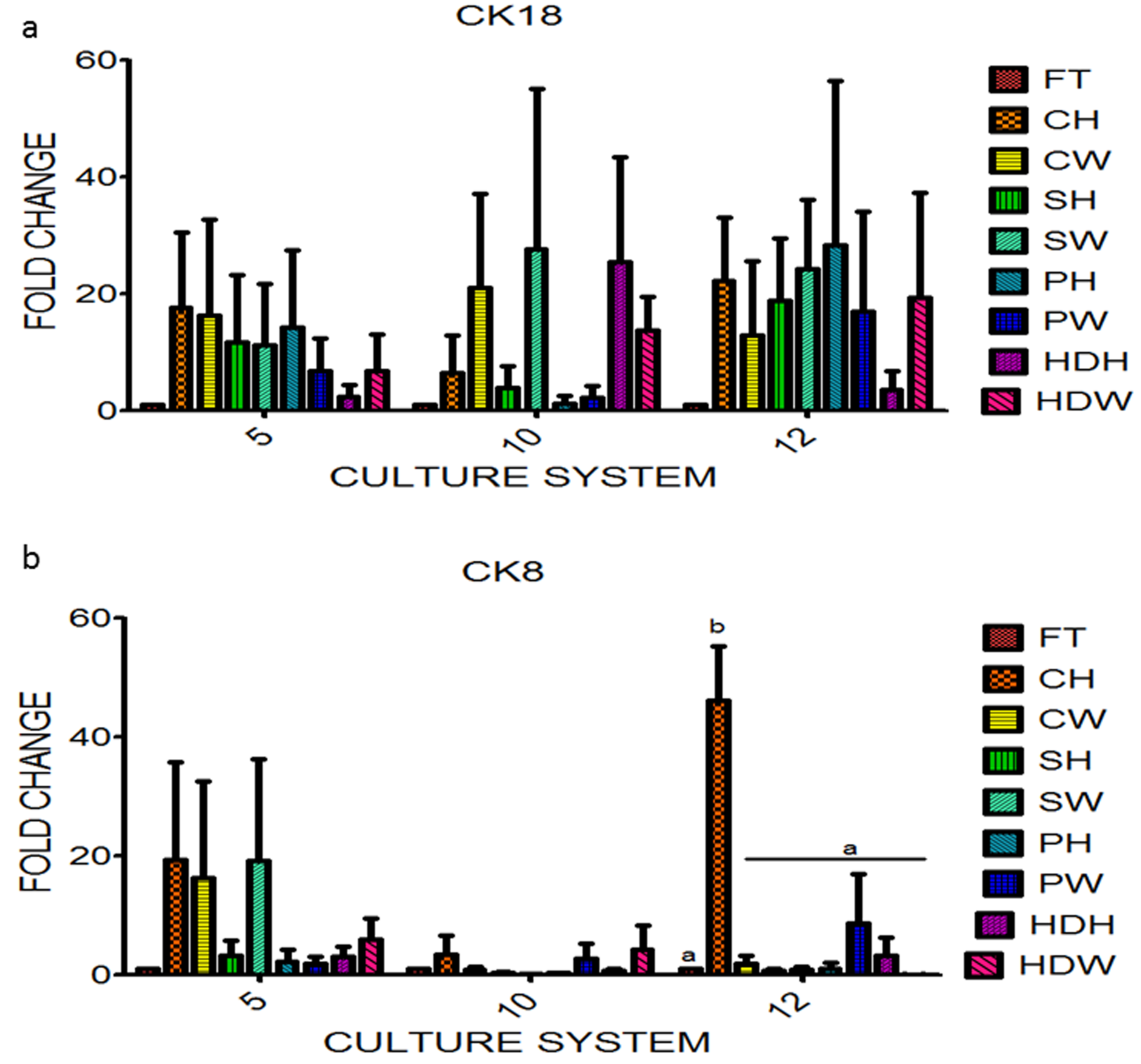

Supplementary Figure S9. Gene expression of the *CK18* (Cytokeratin 18) and *CK8* (Cytokeratin 8) in cultured sheep hepatocytes. 5, 10 and 12 are the culture days. **A.** CK18: There is no significant difference among culture systems by TWO-WAY ANOVA data analysis with BONFERRONI post hoc test at P<0.05. **B.** CK8: alphabets, a and b, represent a significant difference among culture systems by TWO-WAY ANOVA data analysis with BONFERRONI post hoc test at P < 0.05. The effect of the culture system, time and their interaction is significant at P < 0.05. Expression of CK8 is significantly higher in the case of CH on the twelfth day as compared to fresh cells. Each bar represents a mean of three biological and two technical replicates and each error bar shows a standard error mean (SEM).

Supplementary information 1

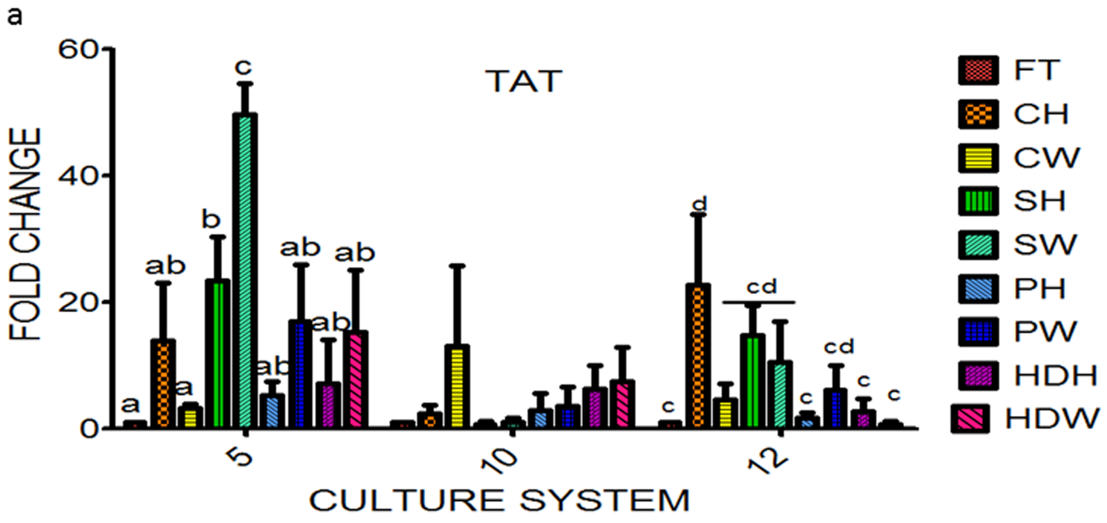

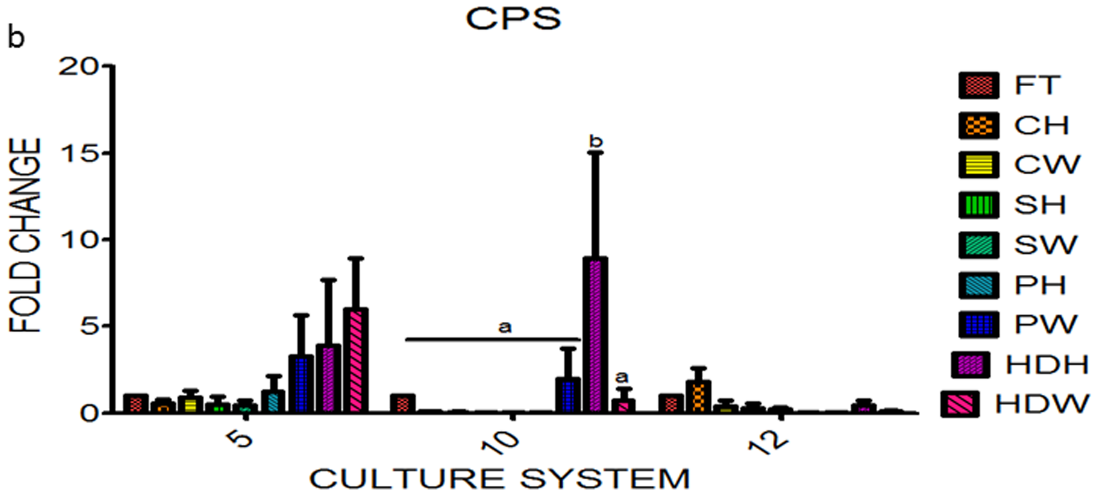

Supplementary Figure S10. Gene expression of the *TAT* (Tyrosine aminotransferase) and *CPS* (Carbamoyl Phosphate Synthetase 1) in cultured sheep hepatocytes. 5, 10 and 12 are culture days. **A.** TAT, the alphabets a, b, c and d represent a significant difference among culture systems by TWO-WAY ANOVA data analysis with BONFERRONI post hoc test at $P < 0.05$. The effects of the culture system, time and their interaction are significant at $P < 0.05$, $P < 0.001$ and $P < 0.01$, respectively. Expression of TAT is significantly higher on the fifth day in SW and on the twelveth day in CH, as compared to fresh cells. **B.** CPS, the alphabets a and b represent a significant difference among culture systems by TWO-WAY ANOVA data analysis with BONFERRONI post hoc test at $P < 0.05$. The effects of the time and the interaction of time and culture systems are significant at $P < 0.05$. The expression of TAT is

significantly higher in the case of HDH on the tenth day as compared to fresh cells. Each bar represents a mean of three biological and two technical replicates and each error bar shows a standard error mean (SEM).

Supplementary information 1

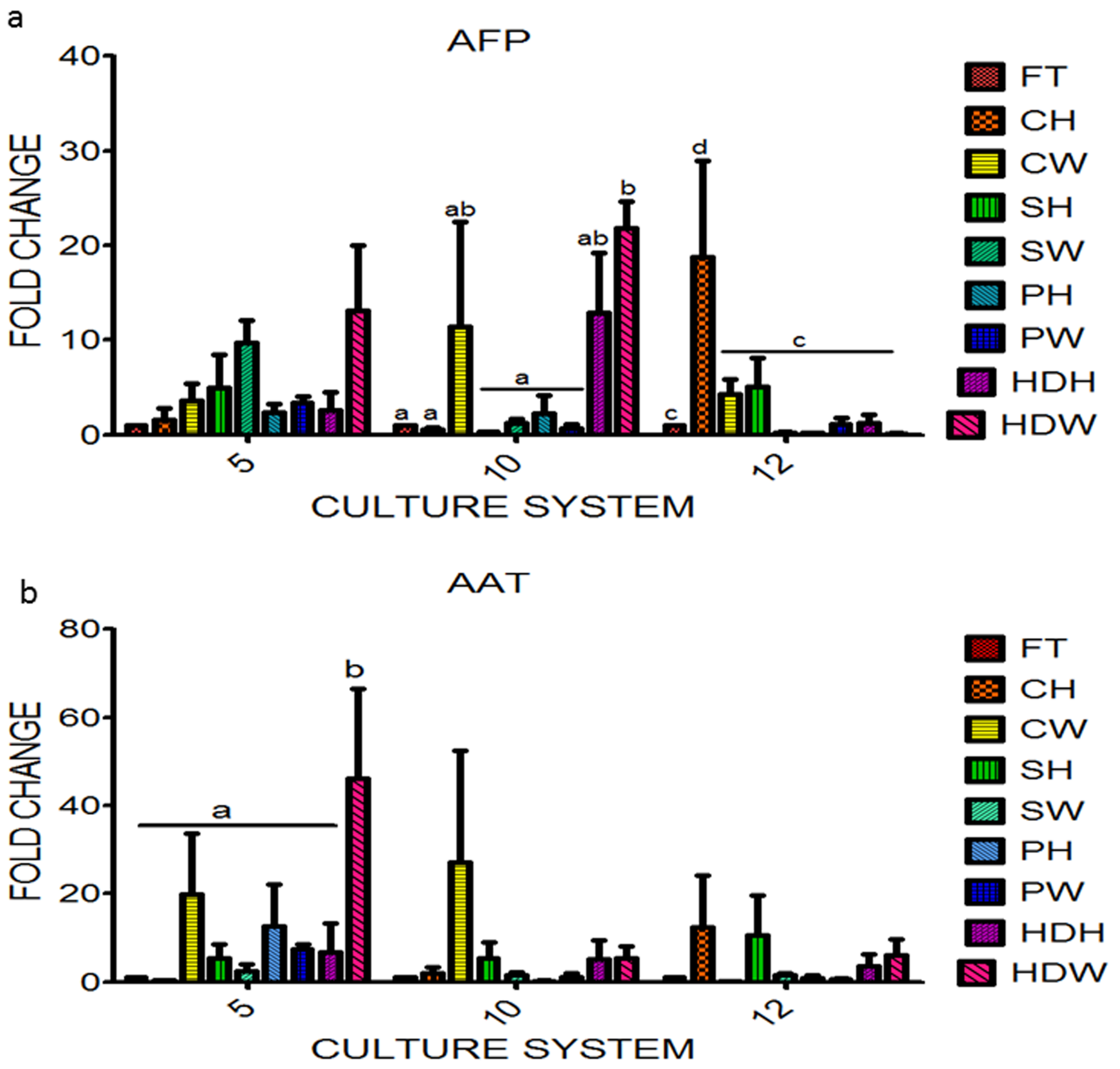

Supplementary Figure S11. Gene expression of the *AFP* (Alpha-Fetoprotein) and *AAT* (Alpha-antitrypsin) in cultured sheep hepatocytes. 5, 10 and 12 are culture days. **A.** AFP, the alphabets a, b, c and d represent a significant difference among culture systems by TWO-WAY ANOVA data analysis with BONFERRONI post hoc test at $P < 0.05$. The effects of the culture system and time are non-signficant. However, their interaction is significant at $P < 0.01$. The expression of the *AFP* is significantly higher in the case of CH on the twelveth day as compared to fresh cells. **B.** AAT, the alphabets a and b represent significant difference among culture systems with TWO-WAY ANOVA data analysis for BONFERRONI post hoc test at $P < 0.05$. The expression of AAT is significantly higher in the case of HDW on the fifth day as compared to fresh cells. Each bar represents a mean of three biological and two technical replicates and each error bar shows a standard error mean (SEM).

Supplementary information 1

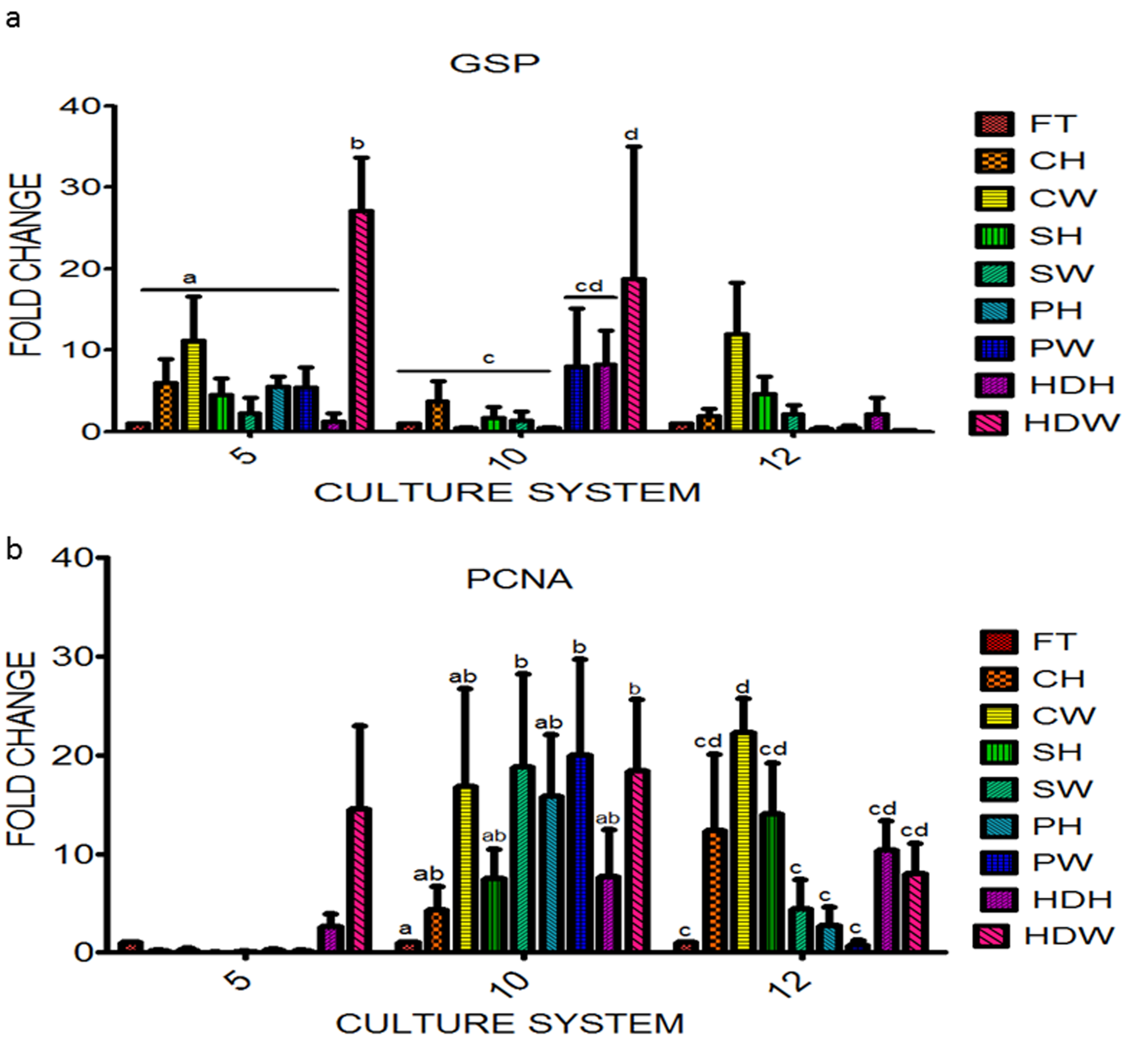

Supplementary Figure S12. Gene expression of the *GSP* (Glucose-6-phoshphatase) and *PCNA* (Proliferative Cell Nuclear Antigen) in cultured sheep hepatocytes. 5, 10 and 12 are culture days. **A.** GSP, the alphabets a and b represent a significant difference among culture systems by TW-WAY ANOVA data analysis with BONFERRONI post hoc test at P < 0.05. The effect of time is significant at P < 0.05. Expression of GSP is significantly higher on the fifthday and the tenth day in the case of CH as compared to fresh cells. **B.** PCNA, alphabets a and b represent a significant difference among culture systems by TWO-WAY ANOVA data analysis with BONFERRONI post hoc test at P < 0.05. The effects of the culture system, time and their interaction are non-significant, significant at P < 0.001 and P < 0.05, respectively. The expression of PCNA is significantly higher in the case of SW, PW and HDW on the tenth day and CW on the

twelfth day as compared to fresh cells. Each bar represents a mean of three biological and two technical replicates and each error bar shows a standard error mean (SEM).

Supplementary information 1

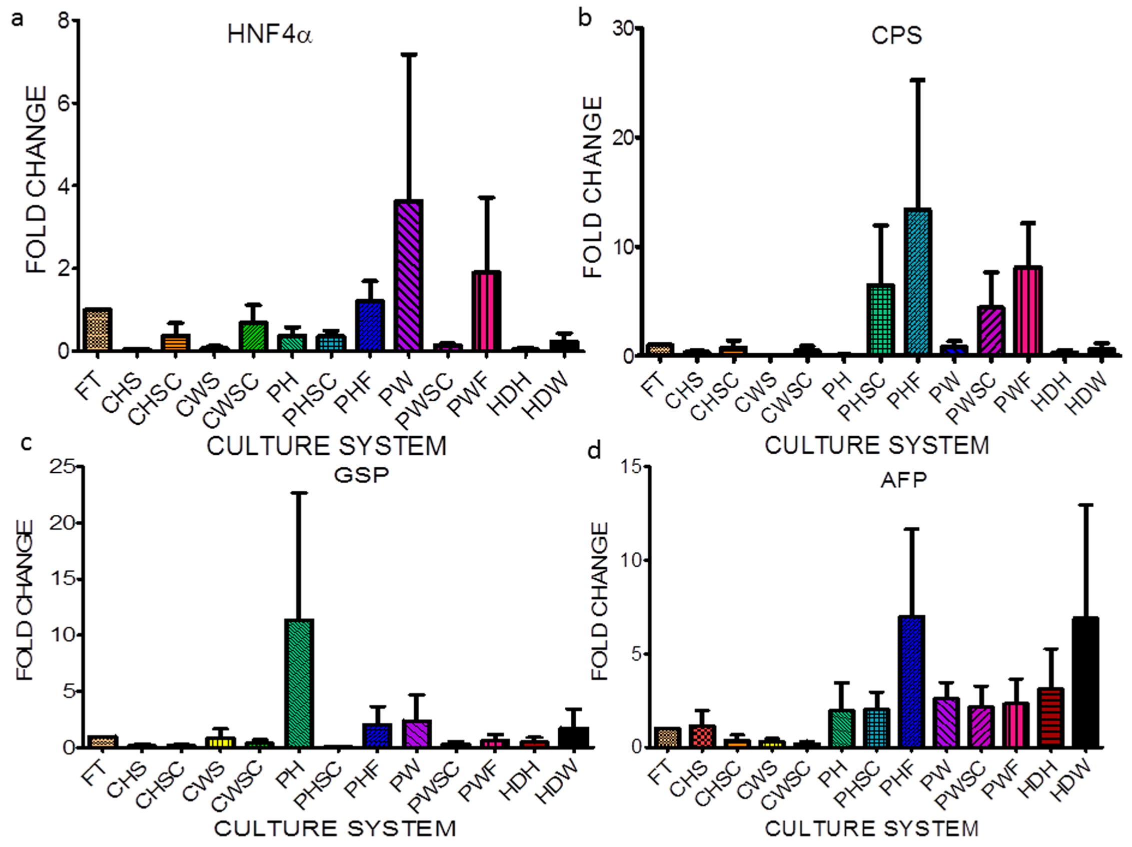

Supplementary Figure 13. Gene expression of the *HNF4α* (Hepatocyte nuclear factor 4 alpha)*, CPS* (Carbamoyl Phosphate Synthetase)*, GSP* (Glucose six phosphatase)*, and AFP* (Alpha-fetoprotein) in cultured buffalo hepatocytes on the sixth day. There is no significant difference among culture systems by ONE-WAY ANOVA data analysis with posthoc Tukey's TEST at $P < 0.05$. **A.** HNF4α. **B.** CPS. **C.** GSP. **D.** AFP. Each bar represents a mean of three biological and two technical replicates and each error bar shows a standard error mean (SEM).

Supplementary information 1

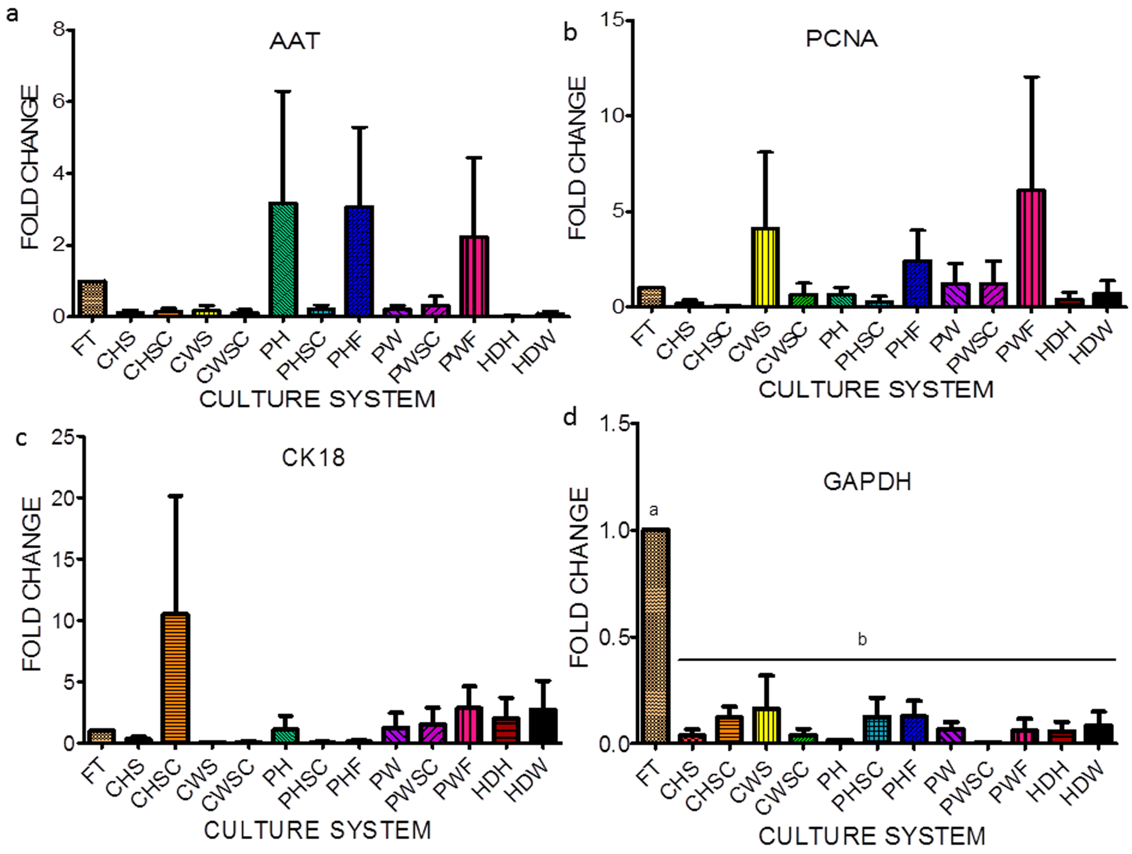

Supplementary Figure 14. The gene expression of the *AAT* (Alpha Anti-Trypsin)*,* *PCNA* (Proliferative Cell Nuclear Antigen)*,* *CK18* (Cytokeratin 18).*,* and *GAPDH* (Glyceraldehyde phosphate dehydrogenase) in cultured buffalo hepatocytes on the sixth day. A. AAT. B. PCNA. **C.** CK18: There is no significant difference among culture systems by ONE-WAY ANOVA data analysis with post hoc Tukey's TEST at P < 0.05. **D.** GAPDH, alphabets a and b represent a significant difference among culture systems by ONE-WAY ANOVA data analysis with post hoc Tukey's TEST at P < 0.05. The expression of GAPDH is significantly lower in all the cultures as compared to fresh cells. Each bar represents a mean of three biological and two technical replicates and each error bar shows a standard error mean (SEM).

Supplementary information 1

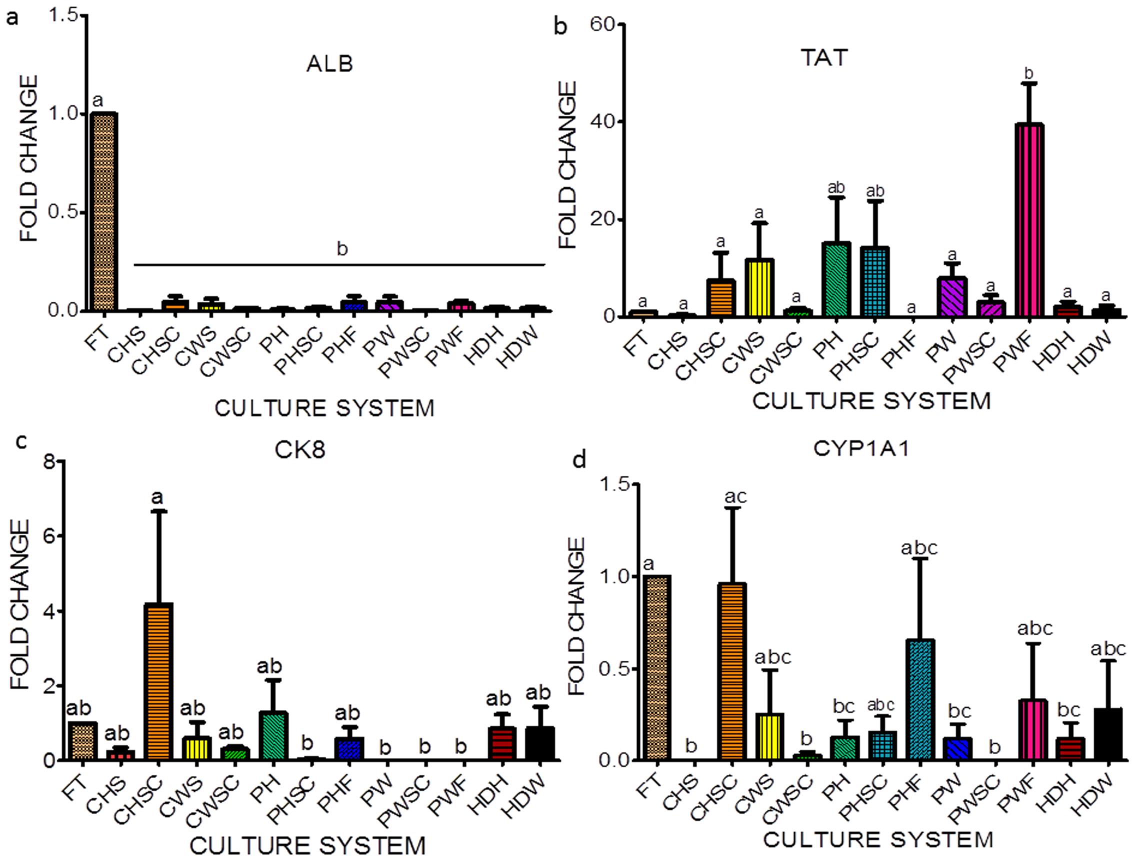

Supplementary Figure 15 The gene expression of the *ALB* (Albumin), *TAT* (Tyrosine Aminotransferase), *CK8* (Cytokeratin 8), and *CYP1A1* (Cytochrome P450, family 1, subfamily A, polypeptide 1) in cultured buffalo hepatocytes on the sixth day. Alphabets a, b and c represent significant difference among culture systems by ONE-WAY ANOVA data analysis with post hoc Tukey's TEST at P<0.05. A. *ALB*. The expression of albumin is significantly lower in all the cultures as compared to fresh cells. **B.** *TAT:* The expression of TAT is significantly higher in the case of PWF as compared to fresh cells. **C.** *CK8:* The expression of the CK8 is significantly lower in the case of PWSC, PHSC, PW and PWF as compared to fresh cells. **D.** *CYP1A1*: The expression of *CYP1A1* is significantly higher in the case of CHS, PWSC, and PWSC as compared to fresh cells. Each bar represents a mean of three biological and two technical replicates and each error bar shows a standard error mean (SEM).
